# Interaction of RNA with a C-terminal fragment of the amyotrophic lateral sclerosis-associated TDP43 reduces cytotoxicity

**DOI:** 10.1038/srep19230

**Published:** 2016-01-13

**Authors:** Akira Kitamura, Yusaku Nakayama, Ai Shibasaki, Ayami Taki, Sachiko Yuno, Kayo Takeda, Masao Yahara, Naoki Tanabe, Masataka Kinjo

**Affiliations:** 1 Laboratory of Molecular Cell Dynamics, Faculty of Advanced Life Science, Hokkaido University, Sapporo 001-0021, Japan; 2 Graduate School of Life Science, Hokkaido University, Sapporo 001-0021, Japan; 3 School of Science, Hokkaido University, Sapporo 001-0021, Japan

## Abstract

A hallmark of amyotrophic lateral sclerosis (ALS), a devastating neurodegenerative disease, is formation of inclusion bodies (IBs) from misfolded proteins in neuronal cells. TAR RNA/DNA-binding protein 43 kDa (TDP43) is an ALS-causative protein forming IBs in ALS patients. The relation between localization of the IBs and neurotoxicity remains largely unknown. We characterized aggregation of fluorescently tagged TDP43 and its carboxyl-terminal fragments (CTFs) by analytical fluorescence imaging techniques. Quantitative time-lapse analysis in individual live cells showed that fluorescent-protein-tagged TDP43 was cleaved and a 35 kDa TDP43 CTF (TDP35) formed ubiquitin (Ub)-negative cytoplasmic IBs. Although TDP35 formed mildly toxic Ub-negative IBs in the cytoplasm, TDP25, another type of a TDP43 CTF, efficiently formed sufficiently toxic Ub-positive IBs. One- or two-color fluorescence correlation spectroscopy (FCS/FCCS) revealed that coaggregation of TDP25 with TDP43 was initiated by depletion of the RNA that binds to TDP25. Moreover, nuclear localization tagging TDP25 reduced the rate of neuronal cell death. These observations point to the need to elucidate the novel sequestration mechanism and details of the toxicity of the misfolded and aggregation-prone TDP43 CTFs (as well as the RNA binding and nuclear retention) in order to identify possible preventive interventions against ALS.

Amyotrophic lateral sclerosis (ALS) is a neurodegenerative disease characterized by dysfunction of motor neurons and by muscle atrophy. Approximately 10% of ALS cases are familial and inherited in an autosomal dominant, autosomal recessive, or X-linked mode; the remaining cases are apparently sporadic^1^,^2^. A common feature of ALS is formation of inclusion bodies (IBs) containing protein aggregates in the cytoplasm and nucleus of motor neurons^3^,^4^. These IBs often contain proteins encoded by ALS-causative genes carrying a mutation. More than 20 proteins have been identified in the ALS-associated IBs, including SOD1, *TARDBP* (TDP43), FUS/TLS, OPTN, and others^5^,^6^,^7^.

The typical feature of ALS-associated proteins is RNA-binding properties, e.g., TDP43 and FUS/TLS^6^. TDP43 is the major disease-associated protein of ALS and frontotemporal lobar degeneration (FTLD-TDP, previously referred to as FTLD-U)^6^. Many ALS-associated missense mutations have been identified in the *TARDBP* gene that cause a substitution of an amino acid^6^,^8^. These TDP43 mutants are intimately involved in the onset and severity of ALS^5^,^9^. TDP43 carries 2 RNA/DNA-recognition motifs (RRM1 and RRM2), which recognize single-stranded (UG)_12_ or (TG)_12_ nucleotide repeats^10^ and a C-terminal glycine-rich region (GRR) including the prion-like Q/N-rich domain (PLD; also called *low complexity sequence domain*; LCD), which regulates interactions with proteins in other RNA-binding proteins *via* a prion-like domain (e.g., hnRNP A1/A2 and FUS/TLS) as well as the self-interaction of TDP43^11^,^12^. TDP43 harbors a nuclear localization signal (NLS) between the N-terminal ubiquitin (Ub)-like domain and RRM1 as well as a nuclear export signal (NES) in RRM2. Thus, TDP43 is subject to nuclear-cytoplasmic shuttling, and the functions of this protein include splicing of mRNA, processing of microRNA, and transport of mRNA to the cytoplasm^6^,^11^,^13^,^14^.

A hyper-phosphorylated and ubiquitinated form of TDP43 accumulates in the IBs in motor neurons of patients with ALS^3^,^15^. In the Ub-positive IB, not only intact TDP43 but also the C-terminal fragments (CTFs) accumulate^3^. TDP43 contains typical DEVD-like motifs in the regions 86–89 and 216–219 in the primary sequence, which are cleaved by caspase 3 (the DETD and DVMD sites, respectively)^16^. The CTFs TDP43_90–414_ (35 kDa) and TDP43_220–414_ (25 kDa) are called TDP35 and TDP25, respectively^17^. A 35 kDa CTF is also alternatively translated from 85^th^ methionine codon^18^. These CTFs are prone to aggregation and form cytoplasmic IBs in cultured cells^17^. Although TDP35 contains the 2 intact RRMs and GRR, TDP25 lacks RRM1 and a portion of RRM2. Misfolded aggregated proteins interfere with cellular functions such as protein folding, protein degradation, and organelle biogenesis (proteostasis/protein homeostasis)^19^,^20^. Thus, the relation between dysregulation of proteostasis by misfolded proteins and modulation of RNA metabolism *via* a loss of function by TDP43 have been implicated in the pathogenesis of ALS^21^.

Aggregating misfolded proteins are partitioned into IBs in the cytoplasm and the nucleus. In the cytoplasm, a perinuclear deposit termed aggresome has been identified^22^. In eukaryotic cells, an aggresome is formed around the microtubule-organizing center (MTOC) during impairment of proteasome activity. Nonetheless, how the cytoplasmic IBs containing TDP43 are formed and the relation between the intracellular partitioning of TDP43-associated IBs and neurotoxicity remain unclear. Here, we demonstrate how IBs containing TDP43 CTFs are formed using biophysical imaging techniques in live cells. Furthermore, we show that prevention of cytotoxic aggregation of TDP25 that takes place after depletion of RNA is potentially involved with nuclear localization.

## Results

### Formation of aggregates from CTFs of TDP43 after cleavage by caspase 3

**Figure 1:**
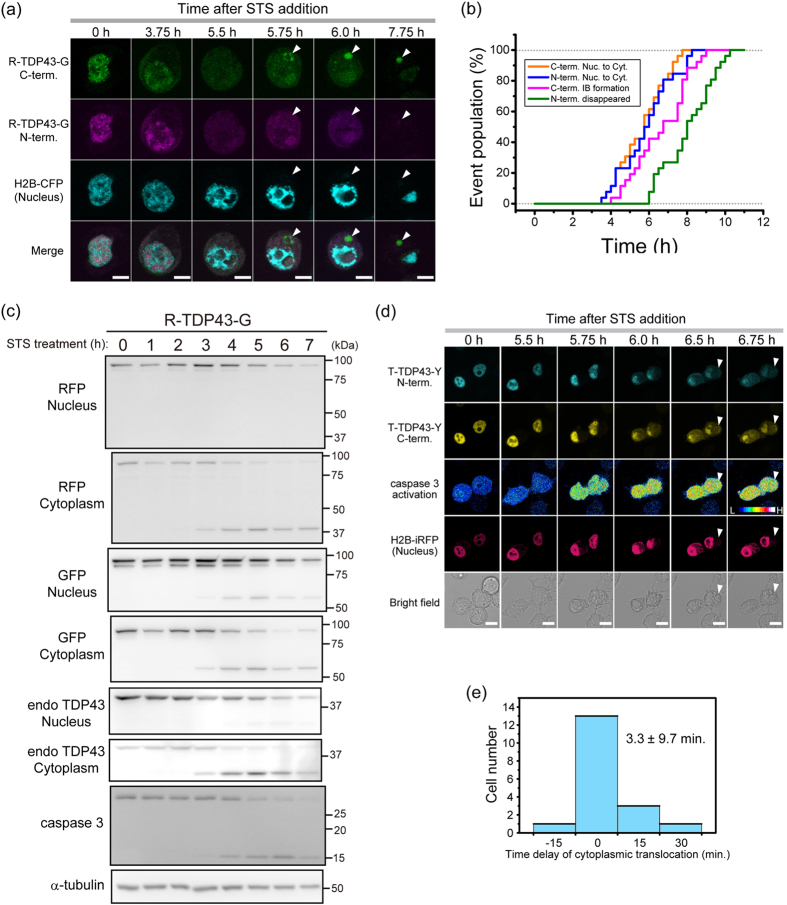
Inclusion body (IB) formation from the carboxyl-terminal fragment (CTF) of TDP43 during activation of caspase 3. (**a**) Image series of the time-lapse analysis during apoptosis. White arrow heads indicate the position of the cytoplasmic IBs of TDP43 CTF. Scale bar = 10 μm. (**b**) Cumulative population of 4 events observed using time-lapse fluorescence microscopy during apoptosis (n = 26). (**c**) A western blot of nuclear and cytoplasmic fractionation of Neuro2A cells expressing R-TDP43-G as well as C termini of endogenous TDP43 during apoptosis. (**d**) H and L in the image of caspase 3 activation denote Förster/Fluorescence resonance energy transfer (FRET) efficiency of the caspase 3 sensor. White arrow heads indicate cytoplasmic IBs. Scale bar = 10 μm. (**e**) A histogram of cells versus the time difference between caspase 3 activation and translocation of the CTF of T-TDP43-Y (n = 18).

Although it is known that caspase 3 cleaves TDP43 and produces a relocation of the TDP43 fragments^16^,^23^,^24^, the detailed process of the cleavage followed by translocation of the fragments into the cytoplasm remains less known. To determine whether CTFs of TDP43 form cytoplasmic IBs or undergo translocation from the nucleus to the cytoplasm in live cells, we used time-lapse fluorescence microscopy to visualize the localization of TDP43 tagged simultaneously with RFP at the N terminus and with GFP at the C terminus (R-TDP43-G) or with mTFP1 at the N terminus and with YFP at the C terminus (T-TDP43-Y) in live murine neuroblastoma cells (Neuro2A) after activation of caspase 3 by incubation with staurosporine (STS; Fig. 1a). At 3.75 h after addition of STS, the N and C termini of R-TDP43-G started to relocate from the nucleus to the cytoplasm, and at 5.5 h, the fluorescence intensity of R-TDP43-G was uniformly distributed throughout the cell (Fig. 1a). The C terminus of R-TDP43-G gradually accumulated in the cytoplasm between time points 5.75 h and 6 h, but the N terminus did not (Fig. 1a), indicating that TDP43 was cleaved and that the accumulated structures were likely IBs. At 7.75 h, the N terminus of R-TDP43-G disappeared from the cell, while the C terminus remained in the cytoplasmic IBs (Fig. 1a). This result indicated that diffusible molecules including N-terminal fragments (NTFs) of TDP43 can leak into the culture medium when the plasma membrane is damaged. Fluorescence recovery after photobleaching (FRAP) analysis revealed that the IBs containing TDP43 CTFs were immobile (Supplementary Fig. 1A). The proportion of the cells in which IBs formed (Fig. 1a) was 11.2% ± 0.4% (mean ± SD; n = 3). This finding suggested that the IBs may be formed only when the accumulation machinery of the aggregate-prone C-terminal fragments in the cytoplasm is functional even in the course of apoptosis.

Next, we quantified those 4 events in a large number of cells during activation of caspase 3 (n = 26, Fig. 1b and Supplementary Fig. 1B,C). Although 50% of the cells showed that the cytoplasmic translocation of the N and C termini of TDP43 occurred within the same period, the remaining cells showed a 15- to 60-min delay of NTF translocation (orange and blue lines in Fig. 1b and Supplementary Fig. 1C, upper left). This result indicates that a portion of TDP43 NTFs were slowly exported from the nucleus. The time point of IB formation was significantly delayed: 59 ± 43.8 min (mean ± SD; n = 26; blue and magenta lines in Fig. 1b and Supplementary Fig. 1C, lower left). This finding suggests that the cytoplasmic localization of TDP43 CTFs after the cleavage may be necessary for IB formation; however, another process in the cytoplasm may trigger the initiation of aggregation of TDP43 CTF. The time point of disappearance of the N termini was significantly delayed: 148 ± 37.7 min (mean ± SD; n = 26), in comparison with the cytoplasmic translocation and IB formation (green line in Fig. 1b and Supplementary Fig. 1C, lower right), indicating that IB formation occurred before the end of the apoptotic process. To characterize the fragments cleaved from R-TDP43-G, we performed biochemical nuclear-cytoplasmic fractionation of the cell lysates expressing R-TDP43-G (99 kDa) after treatment with STS followed by western blotting (Fig. 1c). Emergence of active caspase 3 (15 kDa) and an NTF and a CTF of R-TDP43-G (37 kDa and 62 kDa, respectively) was observed during the same period (Fig. 1c), indicating that TDP43 was cleaved just after activation of caspase 3. Cytoplasmic translocation period of endogenous TDP43 during apoptosis was similar to that of R-TDP43-G as well as cleavage efficiency at 24 h after addition of STS (Fig. 1c and Supplementary Fig. 1D). Previously, TDP25 has been identified as a TDP43 CTF that is produced by caspase 3^17^; however, we observed only a 62-kDa CTF corresponding to TDP35 tagged with GFP on the western blot (Fig. 1c), indicating that caspase 3 promptly cleaved the region between amino acid positions 89 and 90. Moreover, NTFs were not observed in the nuclear fraction, while the C terminus remained. Estimated molecular weight from diffusion property of GFP-TDP35 in the nucleoplasm in live cells was more than 1 MDa (Table 1). These findings also indicated that the TDP43 CTF was retained in the nucleus with formation of large molecular weight complex.

From the observation both nuclear translocation of TDP43 and activation of caspase 3 using a fluorescent sensor^25^, 72% of the cells showed that cytoplasmic translocation of the C terminus of T-TDP43-Y and activation of caspase 3 took place during the same period, and the difference in timing in individual cells was 3.3 ± 9.7 min (mean ± SD; n = 18; Fig. 1e). The cleavage efficiency of T-TDP43-Y was similar to endogenous TDP43 (Supplementary Fig. 1D). These results suggested that the cytoplasmic translocation of the TDP43 fragments starts immediately after the cleavage of TDP43 by caspase 3.

### Distinct compartmentation of TDP43 CTFs

**Figure 2:**
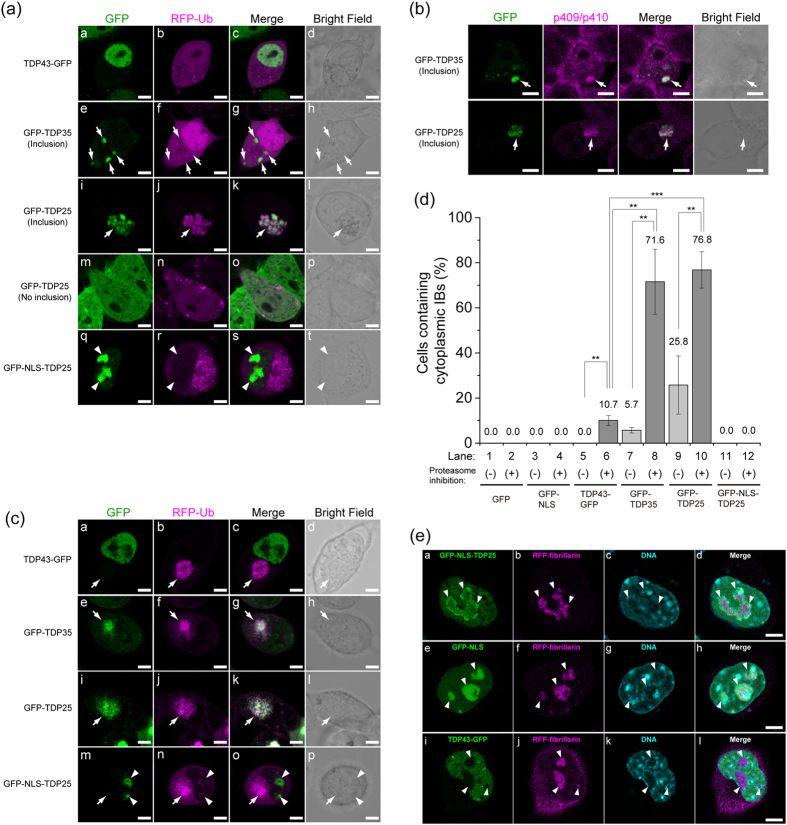
Distinct properties of cytoplasmic and nuclear inclusion bodies (IBs) containing a TDP43 carboxyl-terminal fragment (CTF). (**a**,**c**) Confocal fluorescence microscopy of GFP-tagged TDP43 and TDP43 CTFs during a normal state (**a**), during proteasome inhibition (**c**). The white arrow and arrowhead indicate IBs in the cytoplasm and nucleus, respectively. Scale bar = 5 μm. (**b**) Confocal immunofluorescence microscopy of GFP-tagged TDP35 or TDP25 with an anti-phosphorylated Ser409/410 in TDP43 antibody. Arrowhead indicate IBs in the cytoplasm. Scale bar = 5 μm. (**d**) Quantification and comparison of cytoplasmic IB-positive cells (percentages). The numbers on the bar graph show mean values. The error bars denote mean and SD (n = 3). Light and dark gray mean without (−) and with (+) the proteasome inhibitor MG-132, respectively. (**e**) Airyscan confocal super-resolution microscopy images of TDP43-GFP, GFP-NLS, and GFP-NLS-TDP25. The white arrowhead indicates the position of the nucleolus. Scale bar = 5 μm.

To characterize the IBs including TDP43 CTFs in live cells, we expressed two typical caspase 3-cleaved CTFs tagged with GFP (GFP-TDP35 and GFP-TDP25) in Neuro2A cells and monitored the cells using confocal fluorescence microscopy. GFP-TDP35 or GFP-TDP25 in cytoplasmic IBs was phosphorylated (Fig. 2a,b). IBs containing GFP-TDP25 were ubiquitin-positive (Fig. 2a, i–l), whereas IBs from GFP-TDP35 were not (Fig. 2a, e–h). The distinct ubiquitination state was confirmed using FLAG-TDP35/25 as well as phosphorylation in both of the IBs (Supplementary Fig. 2). TDP43-GFP did not form any obvious IBs in the cytoplasm (Fig. 2d, lane 5). GFP-TDP25 was more prone to aggregation than GFP-TDP35 was, and TDP35 was less polyubiquitinated (Fig. 2a,d). Although Ub-positive IBs containing TDP43 and the CTFs have been identified in the motor neurons of patients with ALS or FTLD^3^,^15^, IBs containing TDP43-GFP after addition of STS for 24 h were less ubiquitinated (Supplementary Fig. 2C). This finding also suggested that a 35 kDa CTF may be less polyubiquitinated.

We did not observe large and distinguishable IBs containing GFP-TDP25 in the nucleus (Fig. 2a, m–p). To test whether the subcellular localization of TDP25 affects the formation of IBs in the cytoplasm, we expressed GFP-TDP25 carrying a Simian-vacuolating-virus-40-derived NLS tag (GFP-NLS-TDP25) in Neuro2A cells to produce an efficient nuclear localization^26^ (Fig. 2a, q–t). GFP-NLS-TDP25 was efficiently localized to the nucleus but also to the nucleolus, as was GFP-NLS as a control (Supplementary Fig. 2D and E), and did not form any cytoplasmic IBs (Fig. 2a, q–t, and Fig. 2d, lane 11). Confocal super-resolution (Airyscan) fluorescence microscopy and three-dimensional reconstruction revealed that GFP-NLS-TDP25 was localized around the fibrillarin (Fig. 2e, a–d; Supplementary Fig. 3A and B; and Supplementary Movie), whereas GFP-NLS was uniformly localized to the nucleolus (Fig. 2e, e–h). Although this nucleolar localization was due to the properties of GFP-NLS^26^, less nucleolar penetration property of GFP-NLS-TDP25 was observed (Supplementary Fig. 2E). This phenomena is likely due to formation of high molecular weight complexes of TDP25 in the nucleoplasm.

Next, during inhibition of proteasome activity using MG-132, GFP-TDP35 as well as GFP-TDP25 was efficiently formed cytoplasmic IBs including ubiquitin (Fig. 2c), as well as increased the number of cells containing cytoplasmic IBs made of TDP43-GFP (10.1% ± 2.1%), GFP-TDP35 (71.6% ± 14.4%), and GFP-TDP25 (76.8% ± 8.1%) as previously reported^17^,^24^,^27^, but we did not observe cells harboring cytoplasmic IBs containing GFP-NLS-TDP25, GFP-NLS, or GFP (Fig. 2d), whereas the proportion of the cells in which GFP-NLS or GFP-NLS-TDP25 was leaked into the cytoplasm (Supplementary Fig. 2D), indicating that TDP43 CTFs can form aggresome structure during dysfunction of proteasome activity as previously reported^28^, however, NLS-tagged TDP25 was avoided from aggresome formation.

**Figure 3:**
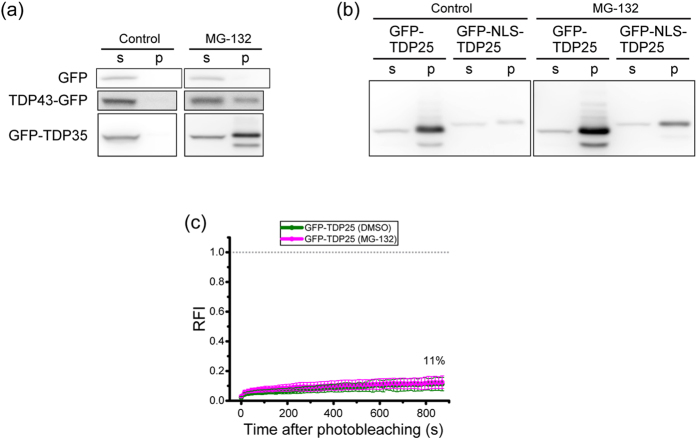
Structural characterization of inclusion bodies (IBs) containing TDP43 carboxyl-terminal fragments (CTFs). (**a**,**b**) Western blotting of GFP-tagged TDP43 and TDP43 CTFs after fractionation into 0.1% SDS soluble (s) or insoluble (p) parts. (**c**) The fluorescence recovery curve of GFP-TDP25 in IBs in the cytoplasm with and without the proteasome inhibitor MG-132 (magenta and green, respectively). The dashed gray line indicates the values of zero and 1.0 of relative fluorescence intensity (RFI). Inset values show the maximum recovery rate.

Next, to determine the solubility of TDP43 and TDP43 CTFs, we performed western blotting followed by detergent-soluble fractionation of the cell lysates. GFP-TDP25 and FLAG-TDP25 was mainly fractionated in the 0.1% SDS-insoluble fraction, whereas the amount of GFP- and FLAG-tagged NLS-TDP25 in the insoluble fraction was low without inhibition of proteasome activity (Fig. 3a,b, and Supplementary Figs 4 and 5). FRAP analysis of GFP-TDP25 in the cytoplasmic IBs showed an immobile accumulation with and without proteasome inhibition (Fig. 3c). Therefore, TDP25 showed heavy accumulation in the insoluble fraction with and without proteasome inhibition, indicating that TDP25 was highly prone to aggregation. Moreover, NLS-tagged TDP25 was less prone to aggregation.

### RNA plays an important role in protection from the formation of aggregates by TDP25

**Figure 4:**
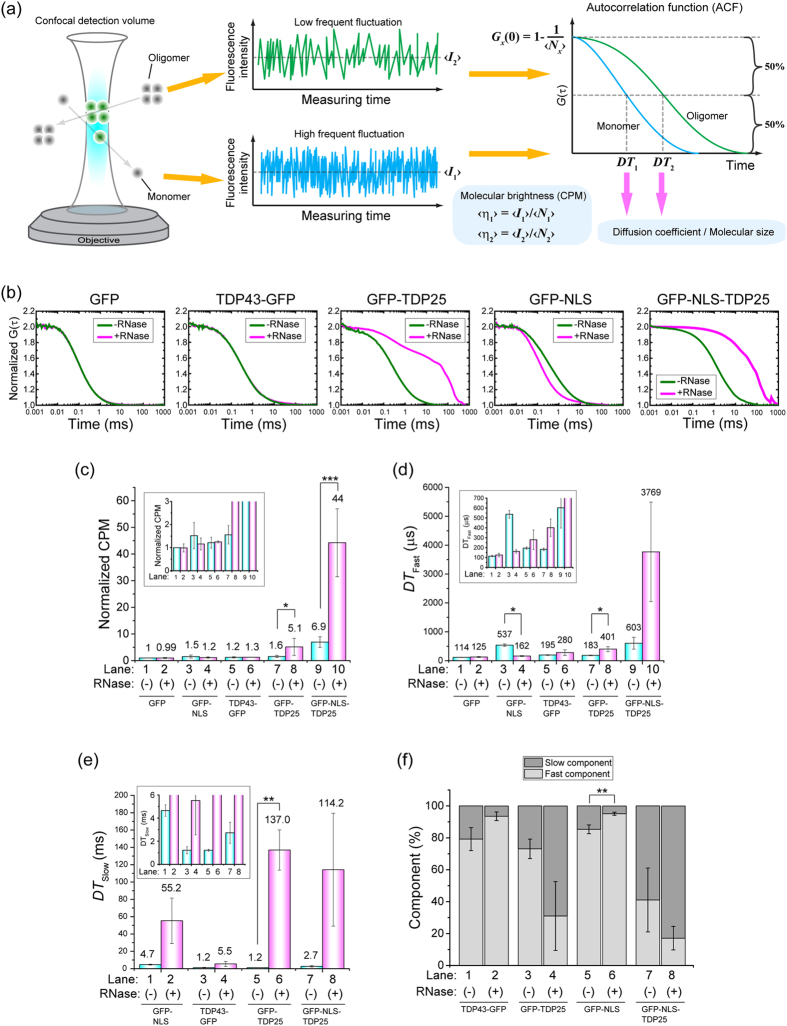
RNase-induced soluble aggregation of TDP25 according to fluorescence correlation spectroscopy (FCS). (**a**) The scheme of measurement and analysis of FCS. <*I*_1_> and <*I*_2_> are average fluorescence intensity of monomers and oligomers, respectively. Diffusion times (*DT*_1_ and *DT*_2_) and the number of molecules (N_x_; *x* = 1 or 2) were obtained from the autocorrelation function (ACF) of the fluctuation. (**b**) Normalized ACFs of GFP-tagged TDP43 and the carboxyl-terminal fragments (CTFs) with (magenta) and without (green) RNase treatment. (C–F) Curve fitting results for ACF. The error bars indicate mean and SD (n = 3). The numbers on the bar graph show mean values. Insets indicates an enlarged view. Significance was tested by Student’s *t* test: *p < 0.05 and **p < 0.01. (**c**) Normalized counts per molecule (CPM) with (magenta) and without (blue) RNase treatment. Values of Fast (**d**) and slow (**e**) diffusion time obtained from curve fitting analysis of a 2-component diffusion model. (**f**) Components of the fast and slow fractions.

We hypothesized that binding of an RNA molecule to aggregation-prone proteins stabilizes the structure of TDP25. To test the effects of RNA depletion on the initiation of aggregation of TDP43 and its CTFs, we performed fluorescence correlation spectroscopy (FCS), which can determine oligomerization and aggregation states of fluorescent molecules *via* analysis of diffusion speed and the molecular brightness per single particle in a cell lysate, with single-molecule sensitivity^29^,^30^ (Fig. 4a). Cells expressing TDP43-GFP, GFP-TDP35, GFP-TDP25, GFP-NLS-TDP25, GFP-NLS, or GFP were lysed and treated with ribonuclease (RNase). Although autocorrelation functions (ACFs) of TDP43-GFP, GFP-TDP35, and GFP were not changed by the treatment with RNase, ACFs of GFP-TDP25 and GFP-NLS-TDP25 were dramatically shifted to the right by this treatment (Fig. 4b, and Supplementary Fig. 6A), indicating that the RNase treatment resulted in slowly diffusing TDP25 species. To quantitatively determine the aggregation state of the molecules, we performed curve fitting for one- and two-component diffusion models of GFP and the other protein, respectively. We focused on counts per molecule (CPM), which indicate average molecular brightness of a single particle. The CPM as well as fast and slow diffusion time (*DT*_Fast_ and *DT*_Slow_, respectively) of GFP-TDP25 and GFP-NLS-TDP25 were significantly increased by the treatment with RNase, whereas CPM of GFP, GFP-NLS, and TDP43 were not (Fig. 4c), indicating emergence of aggregation species of TDP25 by RNA depletion. In contrast, treatment of the cell lysates with DNase I or 0.5 M NaCl did not affect the aggregation state of either GFP-TDP25 or GFP-NLS-TDP25 (Supplementary Fig. 7). Other types of RNase, exonuclease T (3′ → 5′ exoribonuclease activity) or XRN1 (5′ → 3′ exoribonuclease activity), lead to the aggregation of TDP25 (Supplementary Fig. 6B), suggesting that an endogenous RNase may play an important role in the formation of aggregates by TDP25 in live cells. In addition, a gel-shift assay showed that some kinds of RNA can bind to TDP25 (Supplementary Fig. 8). Therefore, these results indicated that RNA depletion specifically caused the soluble aggregation of GFP-TDP25 and GFP-NLS-TDP25. A proteasome inhibitor, MG-132 or epoxomicin, as well as the inhibitors of lysosomal protease did not dramatically affect the RNase-induced aggregation of GFP-TDP25, suggesting that RNA-dependent prevention of GFP-TDP25 aggregation is also maintained during inhibition states of the proteasomal and lysosomal degradation pathway (Supplementary Fig. 6C).

We further analyzed an ALS-associated mutant and the wild type of FUS and SOD1 as another type of an ALS-associated aggregation-prone protein. No ACF shifts were observed in either wild type FUS and SOD1 (FUS^*WT*^ and SOD1^*WT*^) or the ALS-associated mutant of FUS and SOD1 (FUS^*R521G*^ and SOD1^*G85R*^; Supplementary Fig. 6D). This finding indicated that inhibition of oligomerization by RNA was specifically involved in the TDP25-related mechanism.

### Induction of coaggregation of TDP25 with misfolded TDP43 by RNA depletion

**Figure 5:**
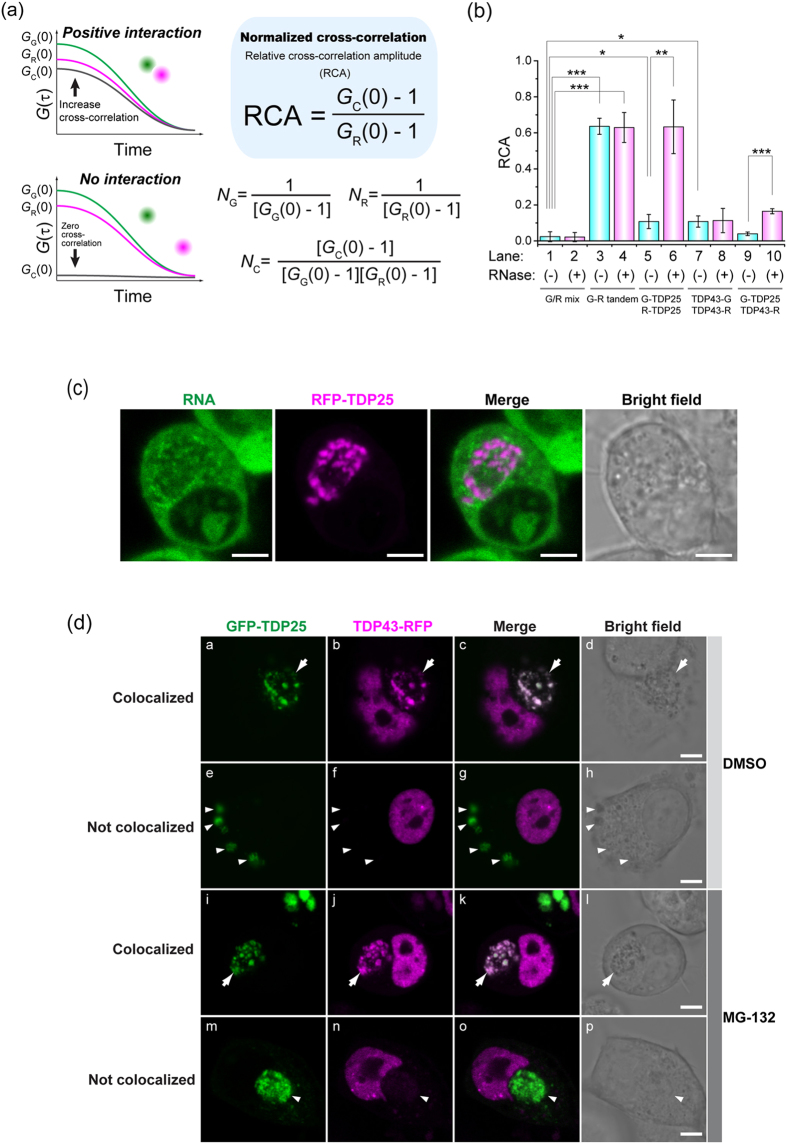
The mechanism of sequestration of TDP43 during TDP25 aggregation. (**a**,**b**) Two-color fluorescence cross-correlation spectroscopy (FCCS). (**a**) A scheme of FCCS analysis and evaluation of interaction strength using relative cross-correlation amplitude (RCA). (**b**) RCA values of the interaction between TDP43 and TDP25 in Neuro2A cell lysate with and without RNase treatment. The error bars indicate mean and SD (n = 3). Significance was tested by Student’s *t* test: *p < 0.05, **p < 0.01, and ***p < 0.001. (**c**) Colocalization of RNA and TDP25 in the cytoplasmic inclusion bodies (IBs) according to confocal fluorescence microscopy. Scale bar = 5 μm. (**d**) Colocalization of GFP-TDP25 with TDP43-RFP in the cytoplasmic IB with and without treatment with a proteasome inhibitor according to confocal fluorescence microscopy. The white arrow shows cytoplasmic IBs in which TDP25 is colocalized with TDP43. The white arrow shows IBs containing TDP25 without TDP43. Scale bar = 5 μm.

To elucidate the detailed mechanism of sequestration of TDP43, we analyzed the state of coaggregation of TDP43 with TDP25 under the influence of RNase treatment by means of two-color fluorescence cross-correlation spectroscopy (FCCS). In this method, which is an advanced version of FCS, intermolecular interaction in solution can be measured directly, with single-molecule sensitivity. In FCCS, relative cross-correlation amplitude (RCA) can estimate the interaction strength^31^ (Fig. 5a). RCA in the cell lysate containing GFP-TDP25 and RFP-TDP25 without treatment with RNase was significantly higher than that of the negative control (0.11 ± 0.040; Fig. 5b, lanes 1 and 5), however, the RNase treatment of the cell lysate containing both GFP-TDP25 and RFP-TDP25 significantly increased RCA: up to 0.63 ± 0.015, but RCA of the RNase-treated cell lysate containing both TDP43-GFP and TDP43-RFP was not increased (Fig. 5b, lanes 6, 7, and 8). This result is confirmation of the TDP25 oligomerization under the influence of depletion of RNA, but this was not the case for TDP43. RCA values of GFP-TDP25 and TDP43-RFP without RNase treatment were similar to RCA of the negative control (0.04 ± 0.01; Fig. 5b, lane 9). Nevertheless, RNase treatment of the lysate significantly increased RCA: up to 0.16 ± 0.014 (Fig. 5b, lane 10), indicating that oligomerized TDP25 after RNA depletion underwent coaggregation with TDP43, but TDP25 and TDP43 barely interacted without the depletion of RNA.

Our FCS and FCCS results raised the question whether TDP25-sequestering IBs can contain RNA. We found that IBs from RFP-TDP25 in the cytoplasm were not colocalized with the RNA-specific fluorescent dye (Fig. 5c and Supplementary Fig. 9), indicating that RNA was not present in the cytoplasmic IBs containing TDP25, and that aggregated TDP25 after depletion of RNA is sequestered into the IBs.

Although TDP43 CTFs, including TDP25, have been identified as components of IBs in neuronal cells^3^, it is unclear what triggers the coaggregation of intact TDP43 with aggregate-prone CTFs. We conducted confocal-microscopy analysis of live cells expressing either GFP-TDP25 or TDP43-RFP. Twenty three percent of the cells harboring IBs containing GFP-TDP25 showed its colocalization with TDP43-RFP (Fig. 5d). Inhibition of proteasome activity increased the number of cells harboring TDP25-positive IBs up to 80.4% (Fig. 2d, lane 10), but the proportion of GFP-TDP25-positive IBs containing TDP43-RFP did not increase (26%, Fig. 5d). In addition, the inhibition of proteasome activity did not dramatically increase the total amount of TDP43 (Fig. 3a). These results suggested that misfolding efficiency of TDP43 may be low, and such protein is immediately degraded at least in healthy cultured cells, but only the misfolded TDP43 is likely to be sequestered with TDP25 during dysregulation of proteostasis.

It was reported that TDP43 recognizes single-stranded stretches of nucleotides with uridine-guanine or thymine-guanine repeats (e.g., UG or TG repeats) *via* the RRM; however, uridine or thymine repeats (U or T repeats) cannot be recognized by TDP43^10^,^32^. We used FCCS to test whether TDP43 and the CTFs can interact with 12-fold UG RNA and TG DNA repeats (UG_12_ and TG_12_) or with 20-mer oligo-U and dT (U_20_ and T_20_) in cell lysate. When either TDP43-GFP or GFP-TDP35 was mixed with UG_12_/TG_12_, a positive amplitude of the cross-correlation function was observed; this was not the case for U_20_/T_20_ (magenta and green in Supplementary Fig. 10), indicating that GFP-TDP35 carries correctly folded RRM and that the protein-oligonucleotide interaction in cell lysates can be analyzed by means of this system. GFP-TDP25, however, showed no positive amplitude of the cross-correlation function for both UG_12_/TG_12_ and U_20_/T_20_ as well as GFP as a control (black, and blue in Supplementary Fig. 10). GFP-NLS-TDP25 and GFP-NLS showed, in contrast to TG_12_ and T_20_, slightly positive cross-correlation for UG_12_ and U_20_, suggesting that this NLS tag weakly binds to RNA as previously reported^26^. This result suggested that the RNA that participated in the prevention of oligomerization/aggregation of TDP25 may be different from the typical TDP43-recognized RNA.

### Cytoplasmic localization of TDP25 results in cytotoxicity

**Figure 6:**
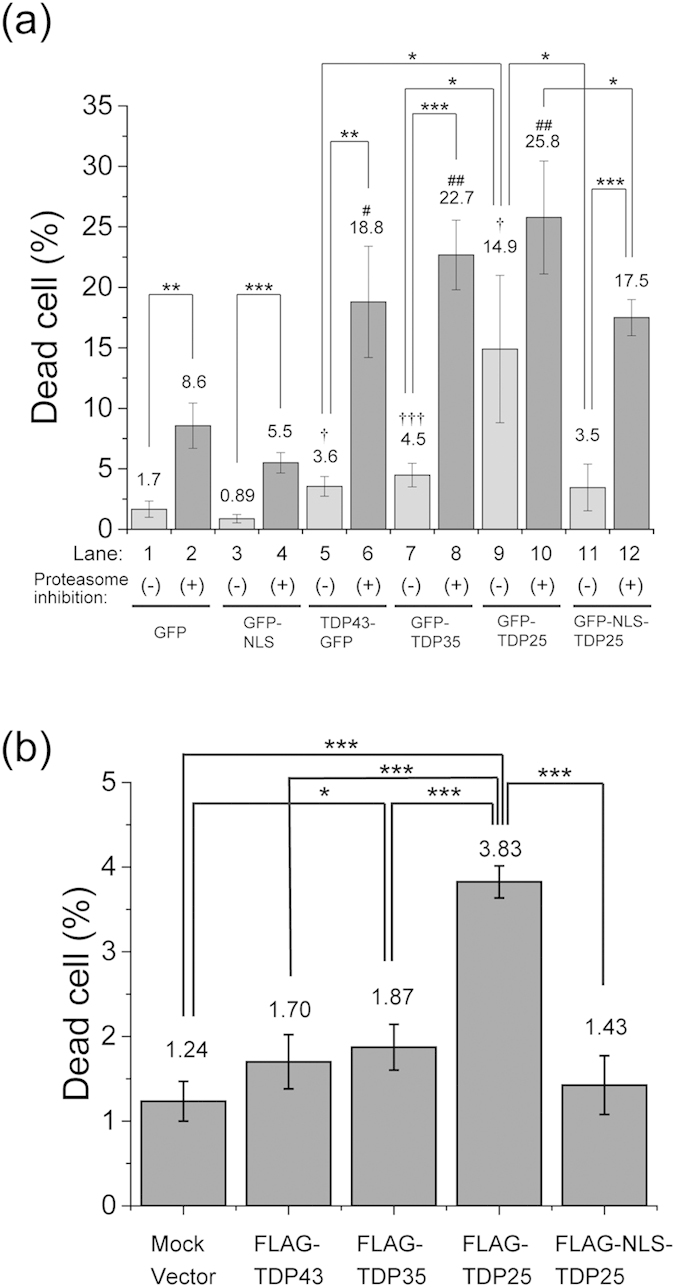
Comparison of the death rate among the Neuro2A cells expressing TDP43 and the carboxyl-terminal fragments (CTFs) with and without treatment with a proteasome inhibitor. Dead cell rate of Neuro2A cells expressing proteins tagged with GFP (**a**) or FLAG (**b**). The error bars indicate mean and SD (n = 3). Significance was assessed by Student’s *t* test. Significance compared to GFP as a negative control without proteasome inhibition is marked as ^†^*p* < 0.05 and ^†††^*p* < 0.001. The significance compared to GFP expression or mock vector transfection as a negative control with proteasome inhibition is marked as ^#^*p* < 0.05 and ^##^*p* < 0.01. Other significance levels are marked as *p < 0.05, **p < 0.01, and ***p < 0.001.

To test how the aggregation predisposition of TDP43 and the CTFs affects cytotoxicity, the numbers of dead Neuro2A cells expressing TDP43 or the CTFs were quantified with and without treatment with a proteasome inhibitor. The proportion of dead cells expressing GFP-TDP25 increased to 14.9% ± 6.1% (Fig. 6a, lane 9). This proportion was significantly higher than that corresponding to TDP43-GFP (3.6% ± 0.81%), GFP-TDP35 (4.5% ± 0.98%), and GFP monomer (1.7% ± 0.67%; Fig. 6a: lanes 1, 5, and 7). These results clearly showed that expression of GFP-TDP25 resulted in the highest cellular toxicity. In contrast, expression of GFP-NLS-TDP25 decreased the proportion of dead cells (3.5% ± 1.9%, Fig. 6a, lane 11). Similar propensities of cytotoxicity were observed using FLAG-TDP43/35/25/NLS25 (Fig. 6b). These results indicate that the NLS-tag inhibits the cytotoxicity of TDP25.

Next, to confirm whether TDP25 just after the cleavage in the nucleoplasm show the less cytotoxicity as well as less aggregation property, nucleoplasmin-derived NLS (NLS^NP^) as another classical NLS was used for TDP25 (GFP-NLS^NP^-TDP25). Less RNA-binding property of GFP-NLS^NP^ was similar to that of monomeric GFP (Supplemental Fig. 11A and B). GFP-NLS^NP^-TDP25 localized only in the nucleoplasm, similarly to localization of GFP-TDP25 in the nucleus (Supplementary Fig. 11C). RNA depletion also induced aggregation of GFP-NLS^NP^-TDP25 (Supplementary Fig. 11D). The GFP-NLS^NP^-TDP25 showed significantly low cytotoxicity compared to GFP-TDP25 (Supplementary Fig. 11E). Moreover, GFP-TDP25 carrying F229/231L mutation (FL) and an RRM2-lacking CTF (GFP-TDPCTF_274–414_ 33) showed similar cytotoxicity as well as aggregation propensity to GFP-TDP25 (Supplemental Fig. 12). Therefore, RNA binding with the likely PLD/LCD in TDP25 reduces cytotoxicity *via* prevention of the aggregation; and nuclear localization may contribute to a role for the reduction of cytotoxicity.

Next, we tested the toxicity during inhibition of proteasome activity. The number of dead cells expressing TDP43-GFP or TDP43 CTFs increased (Fig. 6a, lanes 6, 8, 10, and 12). These numbers were greater than the number of cells expressing the GFP monomer and GFP-NLS (Fig. 6a, lanes 2 and 4). In addition, even during the inhibition of proteasome activity, GFP-NLS-TDP25 showed lower cytotoxicity than did GFP-TDP25 (Fig. 6a, lanes 10 and 12). These results suggested that inhibition of proteasome activity strongly increased vulnerability of the cells to cytotoxic effects of TDP43 or its CTFs; however, the nuclear localization of TDP25 may reduce this cytotoxicity.

## Discussion

We used quantitative time-lapse living cell imaging microscopy and western blotting to clarify the cleavage and transport processes affecting TDP43 after activation of caspase 3. Although these processes were previously reported^16^,^34^,^35^,^36^, we directly found that cytoplasmic translocation of a TDP43 NTF and CTF is initiated immediately after the cleavage by caspase 3 as well as a portion of the CTF forming more than 1 MDa complex with endogenous molecules retained in the nucleus after the cleavage (Fig. 1 and Table 1). Moreover, the TDP43 NTF diffused out of the cells at the end of the apoptotic process; however, some of the TDP43 CTF molecules were retained and formed an immobile structure in the cytoplasm after the cell death (Fig. 1a). Thus the aggregation-prone TDP43 CTF binds to various structures within the cell at the end of apoptosis.

By means of FCS and FCCS analyses, we found that formation of oligomers/aggregates of TDP25 is inhibited by RNA that does not contain a consensus TDP43-recognition sequence, that is, single-stranded UG_12_/TG_12_ nucleotide repeats (Figs 4 and 5, and Supplementary Fig. 10). TDP25 lacks RRM1 and a portion of the RNA/DNA-binding RRM2. The similar region in RRM2 of TDP25 likely does not have good affinity for RNA because other amino acid residues in the correctly folded RRM structure are required for RNA recognition^32^, according to our FCCS results (Supplementary Fig. 10)

**Figure 7:**
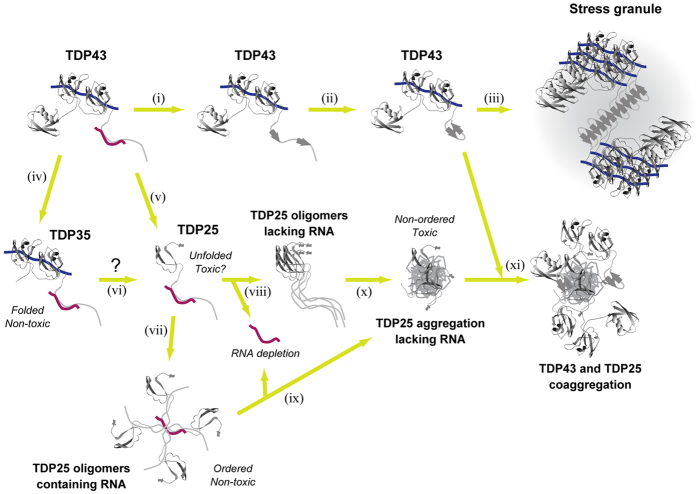
The scheme of the process of aggregation of TDP43 and TDP43 carboxyl-terminal fragments (CTFs). Blue and deep red lines indicate RNA containing TDP43-recognizing (UG) repeats and stabilizing prion-like Q/N rich domain (PLD) of TDP43, respectively. When TDP43 forms stress granules, β-sheet transition of the glycine-rich region (GRR) may be a key for initiation of TDP43 assembly (**i**–**iii**). TDP35 and TDP25 emerge after cleavage of TDP43 (**iv**,**v**). TDP25 may also be produced from TDP35 after further digestion (**vi**). TDP25 forms ordered nontoxic oligomers with RNA (**vii**); however, RNA depletion destabilizes the oligomeric state of TDP25 and results in unordered and toxic TDP25 aggregates (**viii**–**x**). Finally, aggregation of TDP25 sequesters structurally transitioned TDP43 (**xi**). Protein models were generated in Swiss-Pdb viewer v.4.1.0^48^ using Protein Data Bank structures of ubiquitin (ID: 2MJ5)^49^ and RRM1/2 of TDP43 (ID: 4BS2)^10^.

What is the mechanism of inhibition of TDP25 aggregation by RNA? The C-terminal GRR in TDP43 and CTFs contains the disordered PLD or LCDs^37^. The disordered region in the GRR contributes to aggregate formation by TDP43^35^. The disordered PLD in GRR may be stabilized by direct RNA binding to prevent the conformational transition to the aggregated state. It was reported that nucleotides modulate the structure of Prion protein (PrP) and other aggregation-prone proteins^38^,^39^,^40^. Studies of TDP25-stabilizing RNAs and/or RNA-binding proteins to prevent aggregate formation is important to elucidate the regulation mechanism and possible prevention of the prion-like structural transition. One study showed that single-stranded DNA/RNA with UG/TG repeats inhibits insoluble aggregation of TDP43 but not of TDP43 CTF (TDP_208–414_)^41^. Similarly, deprivation of RNA decreases the solubility of TDP43^42^. Although aggregation of intact TDP43 including RRMs may be prevented by the presence of the UG/TG dinucleotide repeats, our study shed light on novel characteristics of the RNA-dependent process of TDP25 aggregation (Fig. 7).

RNA depletion evoked coaggregation of TDP43 by TDP25 (Fig. 5). The function of PLD/LCD is hypothesized to be the reversible assembly of TDP43 in stress granules as well as aggregate formation^43^,^44^. Structural transition of PLD/LCD accelerates the assembly of TDP43 and other stress granule factors. If aggregation-prone proteins containing PLD/LCD (e.g., TDP25) exist, TDP43 carrying a PLD/LCD structure for assembly may be sequestered into the TDP25 aggregation process and result in a loss of function for TDP43. The characteristics of PLD/LCD may be likened to a two-edged sword.

NLS-tagged TDP25 was dramatically downregulated in the SDS-insoluble fraction and stopped the formation of the cytoplasmic Ub- and phosphorylation-positive IBs in Neuro2A cells (Figs 2 and 3). Although GFP-NLS-TDP25 formed ~4 MDa oligomers without RNase treatment, high-molecular-weight aggregates (>740 MDa) emerged after RNase treatment (Fig. 4). The 4-MDa oligomers of GFP-NLS-TDP25 without RNase treatment may disrupt the structural transition and aggregation of TDP25 itself. The inhibition of aggregation by depletion of RNA was also observed in TDP25 tagged with a different type of NLS (GFP-NLS^NP^-TDP25), but not in GFP-NLS^NP^ as a control (Supplementary Fig. 11). Therefore, when RNA binding the NLS region is eliminated, aggregate formation may be synergistically facilitated by the dissociation of TDP25 oligomers *via* NLS and by depletion of the RNA that stabilizes the structure of TDP25. Moreover, the oligomers of GFP-NLS-TDP25 that formed *via* NLS may be in a quasi-stable state.

NLS-tagged TDP25 showed lower cellular toxicity than did TDP25 without the cytoplasmic IB formation (Figs 2 and 6, and Supplementary Fig. 11). These NLS tags are considerably artificial because TDP25 lacks the original NLS sequence, however, the dramatic decrease of cytotoxicity by independent two types of NLS addition may be caused by the nuclear localization as well as the change of oligomeric species of NLS-tagged TDP25. Therefore, cellular toxicity of TDP25 may be facilitated by formation of toxic oligomers/aggregation in the cytoplasm. The toxic oligomers/aggregates of TDP25 may interfere with various functions in the cytoplasm (e.g., cytoskeletal transport and/or organelle homeostasis). Further detailed analysis is needed to clarify the cause of the cytotoxicity in the cytoplasm, as is identification of RNA(s) that would inhibit aggregation of TDP25. The cytoplasmic aggregation of misfolded TDP25 may modulate an imbalance of protein homeostasis and RNA metabolism leading to cytotoxicity. Moreover, inhibition of the structural transition and/or retention of aggregation-prone TDP43 CTFs in the nucleus may retard the development of ALS.

## Methods

### DNA constructs

The following constructs have been described. Human TDP43 cDNA (MGC #3506121; Thermo Fisher Scientific, Waltham, MA) was fused to meGFP^45^, mKate2 (Evrogen, Moscow, Russia), mTFP1 (Allele Biotechnology, San Diego, CA), and mVenus^46^ (gift from Prof. T. Nagai); TDP43-GFP, R-TDP43-G, T-TDP43-Y, respectively. The plasmids harboring EGFP-TDP35 and EGFP-TDP25 (gifts from Dr. Y. J. Zhang and Prof. L. Petrucelli) sequence subcloned into the meGFP-C1 vector^29^ (GFP-TDP35 and GFP-TDP25, respectively). NLS sequences were incorporated into GFP-TDP25 between GFP and TDP25 sequence (GFP-NLS-TDP25 or GFP-NLS^NP^-TDP25). FLAG-tagged TDP43 and the CTFs were modified from GFP-tagged plasmids (FLAG-TDP43/35/25/NLS-TDP25). pBOS-H2B-mSECFP (H2B-CFP) and pBOS-H2B-iRFP (H2B-iRFP) was created using pBOS-H2B-GFP^47^, pRSETb-mSECFP (gift from Prof. T. Nagai), and piRFP (Addgene plasmid #31857). LSSmOrange-DEVD-mKate2 (Addgene plasmid #37132) was used as a sensor for caspase 3-activation. Human ubiquitin sequence (IMAGE #5766897, Thermo Fisher Scientific) subcloned into mCherry-C1 (RFP-Ub). Detailed construction procedures were described in Supplemental Information.

### Antibodies

The following antibodies have been described: anti-GFP (#GF200; Nacalai Tesque, Kyoto, Japan), anti-RFP (#R10367; Thermo Fisher Scientific), anti-caspase 3 (#9662; Cell Signaling Technology, Danvers, MA), an α-tubulin antibody (DM1A; Merck Millipore, Darmstadt, Germany), anti-TDP43 (#3448; Cell Signaling Technology), anti-phosphorylated TDP43 (#1D3; Merck Millipore), anti-Ubiquitin K48 (#Apu2; Merck Millipore), anti-FLAG (M2; Sigma-Aldrich, St. Louis, MO), horse radish peroxidase-conjugated anti-mouse or rabbit IgG (The Jackson Laboratory, Bar Harbor, ME), and AlexaFluor 488/594-conjugated anti-mouse, rabbit, or rat IgG antibody (Thermo Fisher Scientific).

### Cell culture

Mouse neuroblastoma Neuro2A cells were maintained in Dulbecco’s Modified Eagle’s Medium (DMEM; Sigma-Aldrich) supplemented with 10% fetal bovine serum (FBS; GE Healthcare, Logan, UT), 100 U/mL penicillin G (Sigma-Aldrich), and 100 μg/mL streptomycin (Sigma-Aldrich) at 37 °C and 5% CO_2_. Procedures for establishment of stable cell lines were described in Supplemental Information.

### Nuclear-cytoplasmic fractionation

Neuro2A cells transiently expressing R-TDP43-G were lysed at 1 h intervals up to 7 h after the addition of STS (Sigma-Aldrich). After the cells were washed in 2 mL of PBS, 200 μL of hypo-osmotic lysis buffer consisting of 10 mM HEPES-KOH (pH 7.9), 1.5 mM MgCl_2_, 10 mM KCl, 0.1 mM EDTA, 0.1% NP-40, 1 mM DTT, and 1% protease inhibitor cocktail (Sigma-Aldrich) was added, and the mixture was incubated for 5 min at 4 °C. After the lysates were centrifuged at 800 × *g* for 5 min at 4 °C, the supernatants were recovered as a cytoplasmic fraction. Hyperosmotic lysis buffer consisting of 20 mM HEPES-KOH (pH 7.9), 1.5 mM MgCl_2_, 400 mM NaCl, 0.1 mM EDTA, 0.1% NP-40, 10% glycerol, 0.01 U/μL benzonase (Sigma-Aldrich), 1 mM DTT, and 1% protease inhibitor cocktail (Sigma-Aldrich) was added, and the pellets were subjected to shaker conditions at 1,800 rpm for 60 min at 4 °C (#CM-1000; Tokyo Rikakikai Co, Ltd., Tokyo, Japan). After centrifugation at 20,400 × *g* for 5 min at 4 °C, the supernatants were recovered as a nuclear fraction.

### The assay of solubility of TDP43 and the CTFs

Neuro2A cells transiently expressing GFP, TDP43-GFP, GFP-TDP35, GFP-TDP25, or GFP-NLS-TDP25 and these cells under the conditions of the cell viability assay were grown in 3.5-cm NUNC plastic tissue-culture dishes (#150318; Thermo Fisher Scientific). After the cells were washed in 2 mL of PBS, 200 μL of lysis buffer consisting of 25 mM HEPES-KOH (pH 7.5), 150 mM NaCl, 1% NP-40, 1% sodium deoxycholate, 0.1% SDS, 0.01 U/μL benzonase, and 1% protease inhibitor cocktail (Sigma-Aldrich) was added, and the mixture was incubated for 5 min at 4 °C. After the lysates were centrifuged at 20,400 × *g* for 10 min at 4 °C, the supernatants were recovered as a SDS-soluble fraction. The pellets was solubilized in 20 μL of 1 M urea buffered with PBS for 30 min at room temperature followed by washing in 200 μL of PBS. For dilution of urea, 180 μL of PBS was added to the urea-solubilized samples. Each samples were analyzed using western blotting.

### Confocal fluorescence microscopy

Fluorescence observation in live or immunostained cells were examined on an LSM 510 META (Carl Zeiss, Jena, Germany) with a Plan-Neofluar 20×/0.5 NA objective (Carl Zeiss) for time-lapse observation in live cells, a C-Apochromat 40×/1.2 NA UV-VIS-IR Korr. water immersion objective (Carl Zeiss) for high magnitude observation in live cells, or a Plan-Apochromat 63×/1.4 NA DIC oil immersion objective (Carl Zeiss) using immersion oil (#518F, Carl Zeiss) for immunostained cells. Detailed observation condition for each samples were described in Supplemental Information.

### Airyscan (confocal super-resolution) fluorescence microscopy

The immunostained cells were examined on an LSM 880 + Airyscan system (Carl Zeiss) with a Plan-Apochromat 63×/1.4 NA M27 oil immersion objective using immersion oil (#518F, Carl Zeiss) at room temperature. The microscope was operated on the ZEN 2012 software platform (Carl Zeiss). Detailed sample preparation and observation condition were described in Supplemental Information. After calculation of processing for the super-resolution, the images were processed in the ZEN 2012 software and ImageJ 1.47v. Three-dimensional reconstruction of GFP-NLS-TDP25 was performed in the Imaris x64 7.4.2 software (Bitplane, Zurich, Switzerland).

### FRAP

The photobleaching experiments were performed on an LSM 510 META through a C-Apochromat 40×/1.2 NA W Korr. UV-VIS-IR (Carl Zeiss). GFP was excited and photobleached at 488 nm. The photobleaching period was 3.2. Relative fluorescence intensity was measured in the AIM3.2 software (Carl Zeiss) and calculated.

### Fluorescence correlation spectroscopy

Neuro2A cells were lysed in a buffer consisting of 50 mM HEPES-KOH (pH 7.5), 150 mM NaCl, 1% Triton X-100, and 1% protease inhibitor cocktail (Sigma-Aldrich). The supernatant was recovered after centrifugation at 15,780 × *g* for 30 min at 4 °C. After addition of RNase If (50 U/μL), XRN I (1 U/μL), DNase I (6 U/μL), exonuclease T (5 U/μL), NaCl (final 0.5 M), or buffer control, the cell lysates were incubated for 30 min at 25 °C. The cell lysates (20 μL) were applied to Lab-Tek 8-well chamber slides (#155411, NUNC, Rochester, NY) at 25 °C. FCS measurements were performed on a ConfoCor 2 system and C-Apochromat 40×/1.2 NA UV-VIS-IR Korr. water immersion objective (Carl Zeiss) according to a previous study^26^,^29^,^30^. GFP was excited at 488 nm. Confocal pinhole diameter was adjusted to 70 μm. Emission signals were detected through a 505-nm long-pass filter. A multicomponent diffusion model with a triplet state for curve-fitting is given by Eq. 1:


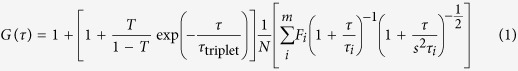


where *G*(τ) is a fluorescence autocorrelation functions, from which the time (τ), *F*_*i*_ and τ_*i*_ are the fraction and diffusion time of component *i*, respectively; *N* is the average number of fluorescent molecules in the analyzed volume defined by the beam waist *w*_0_ and the axial radius *z*_0_; *s* is the structure parameter representing the ratio of *w*_0_ to *z*_0_; *m* is the number of components (*m* = 1 or 2); *T* is the triplet fraction; and τ_triplet_ is the relaxation time of the triplet state. After pinhole adjustment, the diffusion time and structure parameter were determined using a 10^−7^ M rhodamine 6G (Rh6G) solution as a standard before measurements. *G*(τ) in aqueous solutions were measured for 300 s. Other detailed condition for the measurements were described in Supplementary Information.

### FCCS

For analysis of coaggregation between TDP43 and TDP25 under the influence of RNase treatment, Neuro2A cells expressing GFPs and RFPs were lysed and treated with RNase as described in the FCS experiments. FCCS measurements were performed on a ConfoCor 2 system through a C-Apochromat 40×/NA1.2 Korr. UV-VIS-IR water immersion objective (Carl Zeiss). GFP and RFP were excited at 488 and 543 nm, respectively. Detailed condition for the measurements were described in Supplementary Information. RCA values were obtained using Eq. 2, according previously reported^31^.


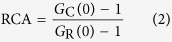


where *G*_C_(0) and *G*_R_(0) are the cross-correlation function and autocorrelation function of DNA at delay time zero, respectively.

For analysis of the interaction between TDP43CTFs and single-stranded oligonucleotides, cell lysate of Neuro2A cells expressing GFP-tagged TDP43 or the CTFs were prepared as described in the other experiments. FCCS analyses were performed on an LSM510 META + ConfoCor3 system through a C-Apochromat 40×/NA1.2 Korr. UV-VIS-IR water immersion objective (Carl Zeiss). Alexa Fluor 647-tagged synthetic single-stranded DNA, TG_12_ or T_20_ oligo-DNA (Life Technologies) or RNA, UG_12_ or U_20_ at 100 nM was added to the cell lysates. GFP and Alexa Fluor 647 were excited at 488 and 633 nm, respectively.

### IB-forming cells and counting of dead cells

Neuro2A cells were transfected with TDP43-GFP and the CTFs tagged with GFP as described in the other experiments and were then cultured for 32 h. After incubation with 2 μM MG-132 or DMSO for 16 h, dead cells were stained with a 1.0 μg/mL propidium iodide (PI) solution (Life Technologies) as described previously^29^,^30^. The percentages of dead and IB-positive cells were calculated from the number of PI-positive cells divided by the number of GFP-positive cells or the number of GFP-positive cells divided by the total number of cells in a visual field, respectively.

### Statistics

To determine statistical significance, Student’s *t* test was performed in Microsoft Excel 2013.

## Additional Information

**How to cite this article**: Kitamura, A. *et al.* Interaction of RNA with a C-terminal fragment of the amyotrophic lateral sclerosis-associated TDP43 reduces cytotoxicity. *Sci. Rep.* **6**, 19230; doi: 10.1038/srep19230 (2016).

## Supplementary Material

Supplementary Information

Supplementary Movie

## Figures and Tables

**Table 1:** Determination of diffusion state of GFP-TDP35 as a mimic of TDP43 CTF by caspase 3 using FCS measurements.

	CPM (kHz)	*D*_Fast_ (μm^2^/s)	Fast component (%)	*D*_Slow_ (μm^2^/s)	Slow component (%)	*M*_w_ from *D*_Fast_ (kDa)	Cell number
GFP-TDP35	3.02 ± 0.396^n.s.^	12.5 ± 2.30^***^	59.7 ± 3.50^***^	0.324 ± 0.756^***^	40.3 ± 3.50^***^	1122	13
GFP	3.28 ± 0.412	43.3 ± 6.74	99.1 ± 0.315	0.0486 ± 0.0545	0.896 ± 0.315	27^†^	8

Each values indicates mean ± S.D. Measured cell number was indicated (Cell number). ^†^Denotes as a basis for calculation of molecular weight from diffusion coefficient. Significance between GFP-TDP35 and GFP was tested by Student’s *t* test: ***p < 0.001 and n.s.: not significant.
