# Supplementary material for: Interaction of RNA with a C-terminal fragment of the amyotrophic lateral sclerosis-associated TDP43 reduces cytotoxicity
Source: Sci Rep. 2016 Jan 13;6:19230. doi: 10.1038/srep19230 (PMC4725827; doi:10.1038/srep19230)

Supplementary Information

Interaction of RNA with a C-terminal fragment of the amyotrophic lateral sclerosis-associated TDP43 reduces cytotoxicity

Akira Kitamura[1], Yusaku Nakayama[1,2], Ai Shibasaki[1,2], Ayami Taki[1,2], Sachiko Yuno[1,2], Kayo Takeda[1,3], Masao Yahara[1,2], Naoki Tanabe[1,3], and Masataka Kinjo[1*]

[1] Laboratory of Molecular Cell Dynamics, Faculty of Advanced Life Science, Hokkaido University, Sapporo 001-0021, Japan

[2] Graduate School of Life Science, Hokkaido University, Sapporo 001-0021, Japan

[3] School of Science, Hokkaido University, Sapporo 001-0021, Japan

* Corresponding author; email: kinjo@sci.hokudai.ac.jp

Materials & Methods

Construction of plasmids

Complementary cDNA sequence of TDP43 (*TARDBP*) was amplified by PCR from a plasmid carrying MGC Collection cDNA (#3506121; Thermo Fisher Scientific, Waltham, MA) as a template, with forward (5′-cgtgaagcttcccaccatgtctgaatatattcgggtaac-3′) and reverse (5′-cgtgggatccgcgtgattcattccccagccag-3′) primers. The amplified fragments were cut and inserted into the meGFP-N1 vector[1]. The sequences of the region encoding TDP43 were confirmed using a genetic analyzer (Applied Biosystems, Waltham, MA), and correct clones were selected (TDP43-GFP). The plasmids harboring EGFP-TDP35 and EGFP-TDP25 sequence[2] were kindly provided by Dr. Zhang Yongjie and Prof. Leonard Petrucelli (Mayo Clinic, Jacksonville, FL). The EGFP sequence of these constructs was replaced with the meGFP sequence *via* Nhe I and Bgl II restriction sites (GFP-TDP35 and GFP-TDP25, respectively). An insert consisting of 3×NLS from Simian vacuolating virus 40[3] or NLS^{NP} from nucleoplasmin[4] (5’-aagcgtccggcagcaaccaagaaagctggccaggctaagaagaagaag-3’) was incorporated into GFP-TDP25 *via* BglII and Hind III restriction sites (GFP-NLS-TDP25). A GFP-coding cDNA sequence (TDP43-GFP or GFP-TDP25) was used to replace mCherry (TDP43-RFP or RFP-TDP25). For establishment of an expression vector encoding TDP43 tagged with both GFP and mKate2 (R-TDP43-G), a TDP43 sequence lacking a start and stop codon was amplified by PCR using forward (5′-cgtgaagcttctgaatatattcgggtaac-3′) and reverse (5′-cgtgggatccgcgtgattcattccccagccag-3′) primers. The amplified fragments were inserted into pmeGFP-N1 *via* Hind III and BamH I restriction

sites, and a clone carrying the correct sequence was selected (start codon-lacking TDP43-meGFP). A fragment of mKate2 cDNA from pmKate2-C1 (Evrogen, Moscow, Russia) was inserted *via* Nhe I and Hind III restriction sites into the start codon-lacking TDP43-meGFP plasmid (R-TDP43-G). A plasmid encoding T-TDP43-Y was constructed by substitution of RFP and GFP in R-TDP43-G in place of mTFP1 and mVenus, respectively. H2B-CFP was created as the GFP sequence for pBOS-H2B-GFP plasmids[5] and was used to replace mSECFP (kindly provided by Dr. T. Nagai, Osaka University, Japan) *via* BamH I and Not I restriction sites. H2B-iRFP was created *via* insertion of iRFP (#31857; Addgene) into pBOS-H2B-GFP in place of the GFP cDNA sequence. A FRET-based caspase 3 indicator (LSSmOrange-DEVD-mKate2)[23] was obtained from Addgene (#37132). Ubiquitin sequence was amplified by PCR using a plasmid containing an IMAGE cDNA (#5766897; Thermo Fisher Scientific) as the template and forward (5′-aagcttcggcccagattttcgtgaaaacccttac-3′) and reverse (5′-ggatccctaaccaccacgaagtctcaacac-3′) primers. The amplified fragment was inserted into the mCherry-C1 vector *via* Hind III and BamH I restriction sites (RFP-Ub). For meGFP expression as a control, cDNA encoding meGFP was inserted into the pTKbX vector (kindly provided by Dr. A. Iwawaki at Gunma University and RIKEN). To obtain a stable cell line, the meGFP, TDP43-GFP, or GFP-TDP25 sequence was inserted into the pcDNA5/TO/FRT plasmid (Thermo Fisher Scientific). A Plasmid coding FUS^{WT}-tagged with Venus was kindly provided from Prof. T. Hirose (Institute for Genetic Medicine, Hokkaido University, Sapporo, Japan). ALS-linked mutant of FUS tagged with Venus (FUS^{R521G}-Venus) was created using PCR.

Cell culture and establishment of a stable cell line

Mouse neuroblastoma Neuro2A cells were maintained in Dulbecco's Modified Eagle's Medium (DMEM; Sigma-Aldrich, St. Louis, MO) supplemented with 10% fetal bovine serum (FBS; GE Healthcare, Logan, UT), 100 U/mL penicillin G (Sigma-Aldrich), and 100 μg/mL streptomycin (Sigma-Aldrich) at 37°C and 5% CO_2. Flp-In TReX HEK293 cells (Thermo Fisher Scientific) stably carrying GFP-TDP25 were selected in DMEM containing 10% FBS, 200 μg/mL Hygro Gold (Invivogen), 5.0 μg/mL Blasticidin S (Invivogen), 100 U/mL penicillin G (Sigma-Aldrich), and 100 μg/mL streptomycin (Sigma-Aldrich) after transfection of pOG44 (Thermo Fisher Scientific) and pcDNA5/TO/FRT plasmids carrying the purpose gene. Flp-In TReX Neuro2A cells[6] (kindly provided from Dr. G. Matsumoto and Dr. N. Nukina, RIKEN, Japan) stably carrying GFP, TDP43-GFP, or GFP-TDP25 were selected in DMEM containing 10% FBS, 200 μg/mL Hygro Gold (Invivogen), 100 μg/mL G418 (Nacalai tesque), 100 U/mL penicillin G (Sigma-Aldrich), and 100 μg/mL streptomycin (Sigma-Aldrich). Stable single clones correctly carrying the genes were selected and expanded. For induction of inserted genes, 1.0 μg/mL doxycycline was added to DMEM containing 10% FBS (GE Healthcare), 100 U/mL penicillin G (Sigma-Aldrich), and 100 μg/mL streptomycin (Sigma-Aldrich).

Time-lapse analysis during apoptosis

Neuro2A cells were grown in a glass-based dish (#3910-035, AGC Techno glass, Shizuoka, Japan) for 16 h before the transfection. A plasmid mixture consisting of 300 ng of the R-TDP43-G-coding plasmid, 400 ng of H2B-CFP, and 300 ng of pCAGGS was transfected into the Neuro2A cells with 2.5 μL of Lipofectamine 2000 (Thermo Fisher Scientific). For detection of caspase 3 activation, the plasmid mixture for transfection was changed to the one consisting of 300 ng T-TDP43-Y, 500 ng LSSmOrange-DEVD-mKate2, and 200 ng H2B-iRFP. At 24 h after the transfection, the medium was replaced with the maintaining medium containing 0.5 μM STS. Immediately after the addition of STS, time-lapse analysis was started on an LSM510 META confocal microscope (Carl Zeiss) through a Plan-Neofluar 20×/0.5NA objective for R-TDP43-G, or a C-Apochromat 40×/1.2NA W Korr. UV-VIS-IR water immersion objective for T-TDP43-Y at 37°C and 5% CO_2. The microscope was operated on the AIM 3.2 software platform (Carl Zeiss). For examination of R-TDP43-G and H2B-CFP, we excited CFP, GFP, or RFP at 458, 488, or 543 nm, respectively. The excitation beams were split by an HFT488/543 filter for GFP and RFP, or an HFT458 filter for CFP. GFP and RFP fluorescent signals were separated by a dichroic mirror (NFT545) and collected through a BP505-530 band pass filter and an LP585 long pass filter, respectively. Fluorescence from CFP was collected through a BP474-525 band pass filter. The pinhole size for CFP, GFP, and RFP was set to 81, 84, and 98 μm, respectively. The zoom factor was set to 1×. X- and Y-scanning sizes were each 512 pixels. For analysis of T-TDP43-Y, LSSmOrange-DEVD-mKate2, and H2B-iRFP, we excited mTFP1, LSSmOrange, and mKate2 at 458 nm, and YFP and iRFP at 514 and 633 nm, respectively. The excitation beams were split by an HFT458 filter for mTFP1, LSSmOrange, and mKate2, by an HFT458/514 filter for YFP, or HFT514/633 filter for iRFP. mTFP1, LSSmOrange, and mKate2 fluorescent signals were simultaneously collected through a spectrophotodetector (META) at 477–499, 584–595, and 659–702 nm, respectively. YFP and iRFP fluorescence was collected through a META at 531–584 and 659–798 nm, respectively. The pinhole size for all conditions was set to 200 μm. The zoom factor was set to 2×. X- and Y-scanning sizes were each 1024 pixels. The acquired images were processed in the ImageJ 1.47v software (National Institutes of Health, Bethesda, MD) and Photoshop CS4 (Adobe Systems, San Jose, CA).

Nuclear-cytoplasmic fractionation and western blotting

Neuro2A cells transiently expressing R-TDP43-G and these cells in the time-lapse condition were grown on a 3.5-cm NUNC plastic tissue culture dishes (#150318; Thermo Fisher Scientific). The cells were lysed at 1 h intervals up to 7 h after the addition of STS. After the cells were washed in 2 mL of PBS, 200 μL of hypo-osmotic lysis buffer consisting of 10 mM HEPES-KOH (pH 7.9), 1.5 mM $MgCl_2$, 10 mM KCl, 0.1 mM EDTA, 0.1% NP-40, 1 mM DTT, and 1% protease inhibitor cocktail (Sigma-Aldrich) was added, and the mixture was incubated for 5 min at 4°C. After the lysates were centrifuged

at 800 × g for 5 min at 4°C, the supernatants were recovered as a cytoplasmic fraction. Hyperosmotic lysis buffer consisting of 20 mM HEPES-KOH (pH 7.9), 1.5 mM $MgCl_2$, 400 mM NaCl, 0.1 mM EDTA, 0.1% NP-40, 10% glycerol, 0.01 U/μL benzonase (Sigma-Aldrich), 1 mM DTT, and 1% protease inhibitor cocktail (Sigma-Aldrich) was added, and the pellets were subjected to shaker conditions at 1,800 rpm for 60 min at 4°C (#CM-1000; Tokyo Rikakikai Co, Ltd., Tokyo, Japan), followed by washing in 200 μL of hypo-osmotic buffer once. After centrifugation at 20,400 × g for 5 min at 4°C, the supernatants were recovered as a nuclear fraction. After 1/3 volume of 4× Laemmli sample buffer was added to each sample, the samples were incubated at 98°C for 2 min. The samples were applied to a 10–20% e-PAGEL (#2331740; ATTO, Tokyo, Japan) and subjected to electrophoresis in SDS-containing buffer. The proteins were blotted on a polyvinylidene difluoride (PVDF) membrane (For GFP, RFP, and tubulin detection, Hybond-P was used instead [purchased from GE Healthcare, Logan, UT]; and for caspase 3 detection, Immobilon-P^{SQ} was used instead; purchased from Merck Millipore, Darmstadt, Germany) using a mini-trans blot cell (Bio-Rad, Hercules, CA). The membranes were blocked in 5% skim milk in PBS-T (PBS containing 0.05% Tween 20). After the membranes were washed in PBS-T 3 times, a primary anti-GFP antibody (GF200; Nacalai Tesque, Kyoto, Japan), anti-RFP (#R10367; Life Technologies), anti-TDP43 antibody (#3448; Cell Signaling Technology, Danvers, MA), anti-caspase 3 (#9662; Cell Signaling Technology, Danvers, MA), or an α-tubulin antibody (DM1A; Merck Millipore) was allowed to react with the membrane in the blocking buffer or Can Get Signal Solution 1 (TOYOBO, Osaka, Japan). As secondary antibodies, anti-mouse or rabbit IgG antibody conjugated with horse radish peroxidase (The Jackson Laboratory, Bar Harbor, ME) was incubated with the membranes in the blocking solution. Images of the luminescent signals were acquired on a LAS 4000 mini (Fujifilm, Tokyo, Japan) with an ECL reagent (GE Healthcare). The images were processed in the ImageJ 1.47v software (National Institutes of Health) and Photoshop CS4 software (Adobe Systems).

The assay of solubility of TDP43 and the CTFs

Neuro2A cells transiently expressing GFP, TDP43-GFP, GFP-TDP35, GFP-TDP25, or GFP-NLS-TDP25 and these cells under the conditions of the cell viability assay were grown in 3.5-cm NUNC plastic tissue-culture dishes (#150318; Thermo Fisher Scientific). After the cells were washed in 2 mL of PBS, 200 μL of lysis buffer consisting of 25 mM HEPES-KOH (pH 7.5), 150 mM NaCl, 1% NP-40, 1% sodium deoxycholate, 0.1% SDS, 0.01 U/μL benzonase, and 1% protease inhibitor cocktail (Sigma-Aldrich) was added, and the mixture was incubated for 5 min at 4°C. After the lysates were centrifuged at 20,400 × g for 10 min at 4°C, the supernatants were recovered as a SDS-soluble fraction. The pellets was solubilized in 20 μL of 1 M urea buffered with PBS for 30 min at room temperature followed by washing in 200 μL of PBS. For dilution of urea, 180 μL of PBS was added to the urea-solubilized samples. After 1/3 volume of 4× Laemmli sample buffer was added to each lysate, the

samples were incubated at 98°C for 5 min. The samples were applied to a 10–20% e-PAGEL (#2331740; ATTO) and subjected to electrophoresis in SDS-containing buffer. The proteins tagged with GFP were detected with an anti-GFP antibody (GF200; Nacalai Tesque), similarly to detection in the nuclear-cytoplasmic fractionation assay.

The assay of cleavage efficiency of TDP43 during apoptosis

Neuro2A cells expressing no transfected plasmids, R-TDP43-G, or T-TDP43-Y were prepared. For STS-untreated cells were trypsinized and suspended in the culturing medium. For STS-treated cells for 24 h, cells were suspended using pipetting. After the centrifugation of the cells, cell pellets were washed in PBS once followed by the pellets were freezed at -80°C. After the pellets were thawed, cells were suspended in PBS containing 1% SDS, 0.01 U/μL benzonase, and 1% protease inhibitor cocktail (Sigma-Aldrich) and then incubated for 10 min. at 25°C. After each soluble fractions were recovered after centrifugation at 20,400 × *g* for 10 min at 25°C, 1/3 volume of 4× Laemmli sample buffer was added to each lysate and then incubated at 98°C for 5 min. The samples were applied to a 10–20% e-PAGEL (#2331740; ATTO) and subjected to electrophoresis in SDS-containing buffer. The proteins were detected with an anti-TDP43 antibody (#3448; Cell Signaling Technology), similarly to detection in the nuclear-cytoplasmic fractionation assay.

Immunofluorescence staining

Neuro2A cells expressing GFP- or FLAG-tagged proteins were fixed in 4% paraformaldehyde buffered with 100 mM Hepes-KOH (pH7.5) at 37°C. After cells were washed in Tris-buffer saline (TBS) three times, PBS containing 0.5% Triton X-100 and 0.5% Saponin was treated for membrane permeabilization. Non-specific binding of antibodies were blocked in PBS containing 5% normal goat serum (DAKO, Glostrup, Denmark) and 10% glycerol (Blocking buffer). Primary and secondary antibodies were reacted in the blocking buffer. Cells were mounted in ProLong Gold (Thermo Fisher Scientific).

Confocal fluorescence microscopy

Neuro2A cells were transfected with GFP-tagged TDP43 or the CTFs as described for other experiments above, except for addition of a plasmid coding 100 ng RFP-Ub or 500 ng TDP43-RFP by means of Lipofectamine 2000. By the addition of a plasmid encoding RFP-Ub or TDP43-RFP, the amount of the plasmid encoding GFP-TDP25 was reduced to normalize the total amount of transfected DNA to 1.0 μg. After incubation for 1 day, the media were replaced with the one containing 2 μM MG-132 (Peptide Institute, Osaka, Japan) or 0.4 μL DMSO (Nacalai Tesque). After incubation for 16 h, confocal fluorescence microscopy was conducted on an LSM 510 META through a C-Apochromat 40×/1.2NA W Korr. UV-VIS-IR (Carl Zeiss) at 37°C and 5% CO_2. The microscope was operated on

the AIM 3.2 software platform (Carl Zeiss). GFP and RFP were excited at 488 or 543 nm, respectively. The excitation beams were split by an HFT488/543 filter. GFP and RFP fluorescent signals were separated by a dichroic mirror (NFT545) and collected through a BP505-530 band pass filter and an LP585 long pass filter, respectively. Pinhole sizes for GFP and RFP were set to 70 and 82 μm, respectively. The zoom factor was set to 4×. X- and Y-scanning sizes were each 1024 pixels. The images were reconstructed using an average value of 4-line scanning. The images were processed in the ImageJ 1.47v software (National Institutes of Health) and Photoshop CS4 software (Adobe Systems).

RNA staining in live cells

Neuro2A cells expressing RFP-TDP25 were stained with medium containing 5 μM SYTO RNA Select (Thermo Fisher Scientific) for several hours. After the cells were washed in Hank's balanced salt solution (HBSS; Sigma-Aldrich), confocal fluorescence microscopy was performed according to the conditions for GFP and RFP analyses. Neuro2A cells expressing GFP-TDP25 were stained in the medium containing 1 mM SYTO62 (Thermo Fisher Scientific) for 30 min. After the cells were washed in HBSS, confocal fluorescence microscopy was conducted as described above for GFP and RFP.

FRAP

The photobleaching experiments were performed on an LSM 510 META through a C-Apochromat 40x/1.2NA W Korr. UV-VIS-IR (Carl Zeiss). GFP was excited and photobleached at 488 nm. GFP fluorescence was collected through a band pass filter (BP505-550). Pinhole sizes were set to 72 μm. The zoom factor was set to 5×. X- and Y-scanning sizes were each 512 pixels. Interval time for image acquisition was set to 15 or 10 s (for cytoplasmic IB or nucleoli, respectively). The photobleaching period was 3.2 or 1.3 s (for cytoplasmic IB or nucleoli, respectively). Relative fluorescence intensity was measured in the AIM3.2 software (Carl Zeiss) and calculated according to Eq. 1.

$$\mathrm{RFI} = \frac{I_{\mathrm{BL}}(t) \cdot I'_{\mathrm{Ref}}}{I'_{\mathrm{BL}} \cdot I_{\mathrm{Ref}}(t)} \qquad [1]$$

where $I_{\mathrm{BL}}(t)$ and $I_{\mathrm{Ref}}(t)$ are the intensity at time point t in the photobleached region and the reference region, respectively. I'_{BL} and I'_{Ref} are the intensity before photobleaching. The recovery curve of relative fluorescence intensity was fitted to Eq. 2 in the Origin 2015 software (OriginLab Corp., Northampton, MA).

$$I(t) = I_0 + Ae^{-t/k} \qquad [2]$$

where $I(t)$ is the intensity at time point t, I_0 is the base line intensity, A is the maximum recovery rate,

and k is recovery constant.

Airyscan (confocal super-resolution) fluorescence microscopy

Both mCherry-fibrillarin (RFP-fibrillarin)[3] and GFP-NLS, GFP-NLS-TDP25, or TDP43-GFP were transfected by means of Lipofectamine 2000 into Neuro2A cells growing in the glass based dishes (#3910-035; AGC Techno glass). At 24 h after transfection, the cells were fixed in 4% paraformaldehyde buffered with 100 mM HEPES-KOH (pH 7.5) for 30 min at 37°C. After the cells were washed in TBS (Santa Cruz) 3 times, 0.2% Triton X-100 in PBS was added for cell membrane permeabilization, with incubation for 5 min at room temperature. The cells were blocked in blocking buffer containing 5% normal goat serum (Wako) and 20% glycerol in PBS for 1 h at room temperature. The mixture of both a rabbit polyclonal anti-GFP antibody conjugated with Alexa Fluor 488 (Thermo Fisher Scientific) and a mouse monoclonal anti-RFP antibody (M165-3; MBL, Nagoya, Japan) in the blocking buffer was used for primary staining. As a secondary staining, we used an anti-mouse IgG antibody conjugated with Alexa Fluor 594. The cells were stained with 1.0 μg/mL Hoechst 33342 (Sigma-Aldrich) in PBS. After we washed out the reagents in PBS, the cells were mounted onto ProLong Gold (Thermo Fisher Scientific). For super-resolution microscopy, the cells were examined on an LSM880 + Airyscan system (Carl Zeiss) with a Plan-Apochromat 63×/1.4NA M27 oil immersion objective using immersion oil (#518F, Carl Zeiss) at room temperature. MBS 405 and MBS 488/561/633 were used as beam splitters. Hoechst33342 was excited at 405 nm and the fluorescent signals were collected through a 420–480 band-pass filter. Alexa Fluor 488 and Alexa Fluor 594 were excited at 488 and 561 nm, respectively, and the fluorescent signals were collected through a 500–545 band pass filter and a 575 long pass filter. Images were acquired at 1200 pixels each. Z-stack series were acquired for 64 stacks. The microscope was operated on the ZEN 2012 software platform (Carl Zeiss). After calculation of processing for the super-resolution, the images were processed in the ZEN 2012 software and ImageJ 1.47v. Three-dimensional reconstruction of GFP-NLS-TDP25 was performed in the Imaris x64 7.4.2 software (Bitplane, Zurich, Switzerland).

Fluorescence correlation spectroscopy

Neuro2A cells were cultured in 3.5-cm plastic dishes (BD Bioscience, Franklin Lakes, NJ) for 16 h before transfection. For expression of GFP, TDP43-GFP, GFP-TDP35, GFP-TDP25, GFP-NLS, and GFP-NLS-TDP25, the amount of plasmid DNA was 200, 400, 1000, 1000, 100, and 1000 ng, respectively. To increase the amount of total plasmid DNA to 1.0 μg, pTKbX was added as necessary. At 24 h after transfection with Lipofectamine 2000, the cells were lysed in a buffer consisting of 50 mM HEPES-KOH (pH 7.5), 150 mM NaCl, 1% Triton X-100, and 1% protease inhibitor cocktail (Sigma-Aldrich). Flp-In T-ReX HEK293 cells stably carrying the *GFP-TDP25* gene were cultured in 3.5-cm plastic dishes (BD Bioscience) in a medium containing 1.0 μg/mL doxycycline for 24 h. The

reagents 2 μM MG-132 (Peptide Institute, Osaka, Japan), 10 nM epoxomicin (Sigma-Aldrich), 10 μg/mL E64d (Sigma-Aldrich), and 10 μg/mL pepstatin A (Sigma-Aldrich), or 0.4 μL of DMSO as a control was added to the medium. HEK293 cells were lysed under the same conditions. The supernatant was recovered after centrifugation at 15,780 × *g* for 30 min at 4°C. After addition of RNase If (50 U/μL), XRN I (1 U/μL), DNase I (6 U/μL), exonuclease T (5 U/μL), NaCl (final 0.5 M), or buffer control, the cell lysates were incubated for 30 min at 25°C. The cell lysates (20 μL) were applied to Lab-Tek 8-well chamber slides (#155411, NUNC, Rochester, NY) at 25°C. FCS measurements were performed on a ConfoCor 2 system and C-Apochromat 40×/1.2NA UV-VIS-IR Korr. water immersion objective (Carl Zeiss) according to a previous study[1, 3, 7]. GFP was excited at 488 nm. Confocal pinhole diameter was adjusted to 70 μm. Emission signals were detected through a 505-nm long-pass filter. The fluorescence autocorrelation functions, $G(\tau)$, from which the time (τ) and the absolute amount of fluorescent proteins in the detection volume were calculated, were obtained according to Eq. 4:

$$G(\tau) = \frac{\langle I(t) \cdot I(t+\tau) \rangle}{\langle I(t) \rangle^2} \qquad [4]$$

where $I(t+\tau)$ is the fluorescence intensity obtained by the single-photon counting method in a detection volume at delay time τ (angular brackets denote ensemble averages). A multicomponent diffusion model with a triplet state for curve-fitting is given by Eq. 5:

$$G(\tau) = 1 + \left[1 + \frac{T}{1-T}\exp\left(-\frac{\tau}{\tau_{\mathrm{triplet}}}\right)\right]\frac{1}{N}\left[\sum_{i}^{m} F_i \left(1+\frac{\tau}{\tau_i}\right)^{-1}\left(1+\frac{\tau}{s^2\tau_i}\right)^{-\frac{1}{2}}\right] \qquad [5]$$

where F_i and τ_i are the fraction and diffusion time of component *i*, respectively; *N* is the average number of fluorescent molecules in the analyzed volume defined by the beam waist w_0 and the axial radius z_0; *s* is the structure parameter representing the ratio of w_0 to z_0; *m* is the number of components (m = 1 or 2); *T* is the triplet fraction; and τ_{triplet} is the relaxation time of the triplet state. $G(\tau)$ values in aqueous solutions were measured for 300 s. After pinhole adjustment, the diffusion time and structure parameter were determined using a 10^{-7} M rhodamine 6G (Rh6G) solution as a standard before measurements. The diffusion coefficients of fluorescent molecules (D_{sample}) were calculated from the published diffusion coefficient of Rh6G, D_{Rh6G} (414 $\mu m^2 s^{-1}$) and the measured diffusion periods of Rh6G under condition (τ_{Rh6G}) and with probe proteins (τ_{sample}), according to Eq. 6:

$$D_{\mathrm{sample}} = \left(\frac{\tau_{\mathrm{Rh6G}}}{\tau_{\mathrm{sample}}}\right) \cdot D_{\mathrm{Rh6G}} \qquad [6]$$

For calculation of molecular weight, we used Eq. 7 according to another study[25, 54],

$$M_{\mathrm{Sample}} = \left(\frac{D_{\mathrm{Sample}}}{D_{\mathrm{GFP}}}\right)^3 \cdot M_{\mathrm{GFP}} \qquad [7]$$

CPM were determined as mean brightness of a measured sample divided by the number of molecules determined by FCS analysis. CPM values were calculated as average fluorescence intensity divided by the number of molecules.

FCCS

For analysis of coaggregation between TDP43 and TDP25 under the influence of RNase treatment, Neuro2A cells expressing GFPs and RFPs were lysed and treated with RNase as described in the FCS experiments. FCCS measurements[8, 9, 10] were performed on a ConfoCor 2 system through a C-Apochromat 40×/NA1.2 Korr. UV-VIS-IR water immersion objective (Carl Zeiss). GFP and RFP were excited at 488 and 543 nm, respectively. GFP and RFP fluorescent signals were separated through NFT610, a dichroic mirror. GFP fluorescence was recorded through BP505-530, a band pass filter. RFP fluorescence was recorded through LP650, a long pass filter. The pinhole was set to 70 μm. Other measurement conditions were similar to the conditions for FCS with RNase treatment. ACFs were fitted to Eq. 5. Cross-correlation functions were fitted to Eq. 8.

$$G(\tau) = 1 + \frac{1}{N}\left[\left(1 + \frac{\tau}{\tau_i}\right)^{-1}\left(1 + \frac{\tau}{s^2\tau_i}\right)^{-\frac{1}{2}}\right] \qquad [8]$$

RCA values for estimation of interaction strength were obtained using Eq. 9[9].

$$\mathrm{RCA} = \frac{G_{\mathrm{C}}(0) - 1}{G_{\mathrm{R}}(0) - 1} \qquad [9]$$

where $G_{\mathrm{C}}(0)$ and $G_{\mathrm{R}}(0)$ are the cross-correlation function and autocorrelation function of DNA at delay time zero, respectively.

For analysis of the interaction between TDP43CTFs and single-stranded oligonucleotides, Neuro2A cells expressing GFP-tagged TDP43 or the CTFs were prepared as described in the other experiments. FCCS analyses were performed on an LSM510 META + ConfoCor3 system through a C-Apochromat 40×/NA1.2 Korr. UV-VIS-IR water immersion objective (Carl Zeiss). Alexa Fluor

647-tagged synthetic single-stranded $(TG)_{12}$ or T_{20} oligo-DNA (Thermo Fisher Scientific) at 100 nM was added to the cell lysates in a chamber glass (#155411; Nunc). Alternatively, Alexa Fluor 647-tagged synthetic single-stranded $(UG)_{12}$ or U_{20} oligo-RNA (Gene Design Inc., Osaka, Japan) was added. GFP and Alexa Fluor 647 were excited at 488 and 633 nm, respectively. The excitation laser beam and fluorescent signals were separated through a beam splitter HFT405/488/543/647. GFP and Alexa Fluor 647 fluorescent signals were collected through NFT635VIS, a dichroic mirror, and then BP505-540 and LP655, respectively. The pinhole was set to 70 μm. The structure parameter and optical settings were adjusted using Rh6G and Cy5 solutions as standards. Cross-correlation function divided by $G_R(0)$ value (Normalized $G_c(\tau)$).

Gel-Shift assay

Flp-In TReX Neuro2A expressing TDP43-GFP, GFP-TDP25, or GFP by addition of doxycycline in the culturing medium for 48 h was lysed according to the experiment of RNase treatment followed by FCS measurement. Concentration of each proteins tagged with GFP was measured using a ConfoCor2 (Carl Zeiss)[10], according to the Eq. 10.

$$C = \frac{N}{\pi^{3/2} w^2 z \cdot N_A} \qquad [10]$$

where, C is a concentration of the GFP-tagged protein, N is the number of fluorescence molecule from FCS measurement ($N = [G(0) - 1]^{-1}$), w is radius of the confocal volume, z is half height of the confocal volume. Here, $\pi^{3/2}w^2z$ is an effective confocal volume. N_A is the Avogadro number. The radius (w) is obtained using the Eq. 11. The z was calculated using structure parameter ($s = z/w$).

$$w^2 = 4D_{\mathrm{Rh6G}} \cdot \tau_{\mathrm{Rh6G}} \qquad [11]$$

where, τ_{Rh6G} is the diffusion time of Rhodamine 6G from FCS measurement, D_{Rh6G} is the diffusion coefficient of Rhodamine 6G (414 $\mu m^2/s$). Each lysates were diluted as 2 nM GFP-tagged proteins was included using lysis buffer. Total RNA was extracted from Neuro2A cells using TRIzol Plus RNA Purification kit (Thermo Fisher Scientific) and the concentration was quantified using a spectrophotometer (Shimadzu, Kyoto, Japan). Total RNA (150 ng) as the same volume to the diluted lysates was added to the lysates, and 4× sample buffer containing 200 mM Hepes-KOH (pH7.9) and 40% Glycerol was subsequently added. Samples were separated using a native-gel comprising 4.5% polyacrylamide and 375 mM Hepes-KOH (pH7.5) in an electrophoresis running buffer comprising 35 mM Hepes and 43 mM imidazole for approximately 3 h (120 V) at 4°C. GFP-tagged proteins were exited at 488 nm and visualized using a Typhoon (GE Healthcare). For confirmation of RNA existence

in the gel, RNA was stained using SYBR Gold (Thermo Fisher Scientific) in the electrophoresis running buffer followed by visualization using a UV transilluminator (ATTO).

IB-forming cells and counting of dead cells

Neuro2A cells were transfected with TDP43-GFP and the CTFs tagged with GFP as described in the other experiments and were then cultured for 32 h. After incubation with 2 μM MG-132 or DMSO for 16 h, dead cells were stained with a 1.0 μg/mL propidium iodide (PI) solution (Thermo Fisher Scientific) for 5 min. Images of GFP and PI channels were captured by means of an LSM 510 META microscope through a Plan-Neofluar 10×/0.3NA objective at 37°C as described previously[1, 7]. Pinhole size was set to the maximum (1000 μm). The GFP- and PI-positive cells were counted in ImageJ 1.47v. The percentages of dead and IB-positive cells were calculated from the number of PI-positive cells divided by the number of GFP-positive cells or the number of GFP-positive cells divided by the total number of cells in a visual field, respectively.

Statistics

To determine statistical significance, Student's *t* test was performed in Microsoft Excel 2013.

Supplementary References

1. Kitamura A, *et al.* Dysregulation of the proteasome increases the toxicity of ALS-linked mutant SOD1. *Genes to Cells* **19**, 209-224 (2014).

2. Zhang YJ, *et al.* Aberrant cleavage of TDP-43 enhances aggregation and cellular toxicity. *Proceedings of the National Academy of Sciences of the United States of America* **106**, 7607-7612 (2009).

3. Kitamura A, Nakayama Y, Kinjo M. Efficient and dynamic nuclear localization of green fluorescent protein via RNA binding. *Biochemical and Biophysical Research Communications* **463**, 401-406 (2015).

4. Mattaj IW, Englmeier L. Nucleocytoplasmic transport: the soluble phase. *Annual Review of Biochemistry* **67**, 265-306 (1998).

5. Kanda T, Sullivan KF, Wahl GM. Histone-GFP fusion protein enables sensitive analysis of chromosome dynamics in living mammalian cells. *Current Biology* **8**, 377-385 (1998).

6. Matsumoto G, Wada K, Okuno M, Kurosawa M, Nukina N. Serine 403 phosphorylation of p62/SQSTM1 regulates selective autophagic clearance of ubiquitinated proteins. *Molecular Cell* **44**, 279-289 (2011).

7. Kitamura A, *et al.* Cytosolic chaperonin prevents polyglutamine toxicity with altering the aggregation state. *Nature Cell Biology* **8**, 1163-1170 (2006).

8. Mikuni S, *et al.* Screening for FtsZ Dimerization Inhibitors Using Fluorescence Cross-Correlation Spectroscopy and Surface Resonance Plasmon Analysis. *PloS one* **10**, e0130933 (2015).

9. Saito K, Wada I, Tamura M, Kinjo M. Direct detection of caspase-3 activation in single live cells by cross-correlation analysis. *Biochemical and Biophysical Research Communications* **324**, 849-854 (2004).

10. Tiwari M, Mikuni S, Muto H, Kinjo M. Determination of dissociation constant of the NFkappaB p50/p65 heterodimer using fluorescence cross-correlation spectroscopy in the living cell. *Biochemical and Biophysical Research Communications* **436**, 430-435 (2013).

Supplementary Figure legends

Supplementary Figure 1.
Detailed quantification and characterization of the events including cytoplasmic translocation of TDP43 and formation of inclusion bodies (IBs) according to time-lapse analysis.
(A) Relative fluorescence intensity (RFI) versus before photobleaching in the cytoplasmic IB of C-terminal fragments from R-TDP43-G after the treatment with staurosporine for 24 h according to fluorescence recovery after photobleaching (FRAP; mean ± SD; n = 3). Blue and orange lines showed with and without photbleaching, respectively. (B) Timing of the events including the start of translocation of the N terminus from R-TDP43-G, translocation start of the C terminus from R-TDP43-G, IB formation start from the C terminus of R-TDP43-G, and disappearance of N terminal signals. The numbers on the bar graph show mean values. Error bars indicate SD. Triplicate experiments were performed independently (n = 3). (C) Histograms of the time delay between 2 events in a single cell (total cell number was 26). (D) Caspase 3-cleaved efficiency of endogenous TDP43, R-TDP43-G, and T-TDP43-Y. Arrow and arrowhead showed intact and cleaved TDP43, respectively.

Supplementary Figure 2.

Characterization of FLAG-tagged TDP43 and the CTFs.

(A) Confocal fluorescence microscopy of immunostained cells using FLAG and poly-ubiquitin. Scale bars = 5 μm. (B) Confocal fluorescence microscopy of Ser409/410 phosphorylated cytoplasmic IBs containing FLAG-TDP35/25. Scale bars = 5 μm. (C) Confocal fluorescence microscopy of TDP43-GFP and RFP-ubiquitin after the treatment with 0.5 μM staurosporine for 24 h. Scale bars = 5 μm. (D) Analysis of the cells with cytoplasmic leakage of GFP-NLS and GFP-NLS-TDP25 with and without treatment with the proteasome inhibitor MG-132. The error bars denote mean and SD (n = 4). The significance was analyzed by Student's *t* test (*$p < 0.05$ and ***$p < 0.001$). (E) Analysis of the cells harboring nucleolar localization of GFP-NLS and GFP-NLS-TDP25 with and without treatment with MG-132. The error bars denote mean and SD (n = 4). The significance was analyzed by Student's *t* test: *$p < 0.05$ and **$p < 0.01$.

Supplementary Figure 3.

Three dimensional reconstruction of GFP-NLS-TDP25 using a confocal airyscan super-resolution microscopy.

Airyscan confocal super-resolution microscopy images of GFP-NLS-TDP25. (A) Three-dimensional orthogonal images of a Neuro2A cell expressing GFP-NLS-TDP25. Scale bar = 10 μm. (B) Three-dimensional reconstruction of the super-resolution Z-stack images of GFP-NLS-TDP25 shown in (Figure 2E, a-d). Scale bar = 1 μm.

Supplementary Figure 4.

Comparison of detergent solubility of TDP43-GFP and GFP-TDP25.

Neuro2A cells expressing GFP, TDP43-GFP, or GFP-TDP25 were lysed in 1% Triton X-100 buffer containing 50 mM HEPES-KOH (pH 7.5) and 150 mM NaCl or in 0.1% SDS buffer containing PBS. After centrifugation at 20,400 × *g* for 15 min, supernatants were collected as a soluble fraction (s). The pellets were solubilized by sonication in Laemmli sample buffer (p). Proteins were immunostained with an anti-GFP antibody (GF200, Nacalai Tesque, Kyoto, Japan) or anti-β-actin antibody (Clone #C4, Merck-Millipore).

Supplementary Figure 5.

Comparison of detergent solubility of FLAG-tagged TDP43, TDP35, TDP25, and NLS-TDP25.

Neuro2A cells expressing FLAG-tagged proteins were lysed in a lysis buffer containing 0.1% SDS. After centrifugation at 20,400 × *g* for 15 min, supernatants were collected as a soluble fraction (S). The pellets were solubilized by 1M Urea-PBS (P). Proteins were immunostained with an anti-FLAG

antibody (M2, Sigma-Aldrich).

Supplementary Figure 6.

Confirmation of formation of TDP35 or TDP25 aggregates under the influence of RNase treatment.

(A) Normalized autocorrelation functions (ACFs) of lysates of Neuro2A cells expressing GFP-TDP35 with and without RNase I_f (magenta and green, respectively). (B) Normalized ACFs of lysates of Neuro2A cells expressing GFP (a), TDP43-GFP (b), or GFP-TDP25 (c) after treatment with exonuclease T or XRN1. (C) Normalized ACFs of lysates of HEK293 cells stably carrying the *GFP-TDP25* gene after treatment with a proteasome inhibitor (MG-132 or epoxomicin) or lysosomal protease inhibitors (E64d + pepstatin A). (D) Normalized ACFs of lysates of Neuro2A cells expressing wild type superoxide dismutase 1 (SOD1) (a), the amyotrophic lateral sclerosis (ALS)-associated G85R mutant (b) of SOD1, wild type FUS (c), or the ALS-associated R521G mutant of FUS (d) with and without RNase I_f treatment (magenta and green, respectively).

Supplementary Figure 7.

Results of fluorescence correlation spectroscopy (FCS) of TDP43 and the carboxyl-terminal fragments (CTFs) after treatment with DNase or 0.5 M NaCl.

The error bars denote mean and SD (n = 3). (A–D) Results of DNase treatment. (E–F) Results of NaCl treatment. Comparison of counts per molecule (CPM; panels A and E), fast diffusion time (DT_{Fast}; panels B and F), slow diffusion time (DT_{Slow}; panels C and G), and fast and slow components (D and H). No significant differences were observed.

Supplementary Figure 8.

RNA binding ability of GFP, TDP43-GFP, and GFP-TDP25 using gel-shift assay.

(A) A gel image including GFP, TDP43-GFP, and GFP-TDP25 with or without total RNA. (B and C) A quantification of electrophoretic mobility of GFP-TDP25 (B) or GFP (C) with and without RNA (magenta and green, respectively).

Supplementary Figure 9.

Oligonucleotide staining in a live Neuro2A cell expressing GFP-TDP25.

After the cells were stained with 1 mM SYTO62 (Life Technologies) for 30 min, they were examined under a confocal fluorescence microscope. Bright structures in the green image are the cytoplasmic inclusion bodies (IBs) of GFP-TDP25. Scale bar = 5 μm.

Supplementary Figure 10.

Analysis of single-stranded RNA/DNA recognition by TDP43 and by its carboxyl-terminal fragments (CTFs) by means of fluorescence cross-correlation spectroscopy (FCCS).

Cross-correlation functions were normalized using the value of the autocorrelation function of red fluorescence when $\tau = 0$. The Y-intercepts [$G_c(0)$] of normalized $G_c(\tau)$ correspond to relative cross-correlation amplitude (RCA) values in Figure 5A. (A-D) The normalized cross-correlation function of GFP-tagged TDP43/35/25 and Alexa Fluor-UG_{12} (A), Alexa Fluor-U_{20} (B), Alexa Fluor-TG_{12} (C), and Alexa Fluor-T_{20} (D).

Supplementary Figure 11.

Characterization of GFP-TDP25 including nucleoplasmin-derived NLS.

(A) Normalized cross-correlation function from FCCS measurement in case of Alexa Fluor 647-UG_{12} (left) or U_{20} (right). GFP-NLS^{SV40} is described as GFP-NLS in the main text (B) Comparison of diffusion state of GFP, GFP-NLS^{SV40}, or GFP-NLS^{NP} in cell lysate. (C) Nuclear localization pattern of GFP-NLS^{NP} or GFP-NLS^{NP}-TDP25 using confocal fluorescence microscopy. Scale bar = 5 μm. (D) RNase If treatment in the cell lysate expressing GFP-NLS^{NP} or GFP-NLS^{NP}-TDP25. (E) Dead cell rate of Neuro2A cells expressing GFP, GFP-TDP25, or GFP-NLS^{NP}-TDP25 (mean ± S.D., n=3; $**p<0.01$ and $***p<0.001$)

Supplementary Figure 12.

Cytotoxicity of TDP25 carrying F229/231L mutation and TDP43CTF (274-414).

(A) Cell death rate of Neuro2A cells expressing GFP (Control), GFP-TDP25, GFP-TDP25 carrying F229/231L mutation (TDP25FL), or GFP-$TDP43CTF_{274\text{-}414}$ (CTF274-414) (mean ± S.D., n=3; $*p<0.05$, $***p<0.001$). (B) Expression and solubility check of GFP-TDP25, GFP-TDP25-FL, and GFP-$TDP43CTF_{274\text{-}414}$. Neuro2A cells expressing FLAG-tagged proteins were lysed in a lysis buffer containing 0.1% SDS. After centrifugation at 20,400 × *g* for 15 min, supernatants were collected as a soluble fraction (S). The pellets were solubilized by 1M Urea-PBS (P). Proteins were immunostained with an anti-GFP antibody (GF200, Nacalai tesque).

Supplementary Movie.

Three-dimensional reconstruction of confocal super-resolution (Airyscan) fluorescence image of a Neuro2A cell expressing GFP-NLS-TDP25.

GFP-NLS-TDP25 and RFP-fibrillarin were indicated green and magenta color, respectively.

Supplemental Figure 1

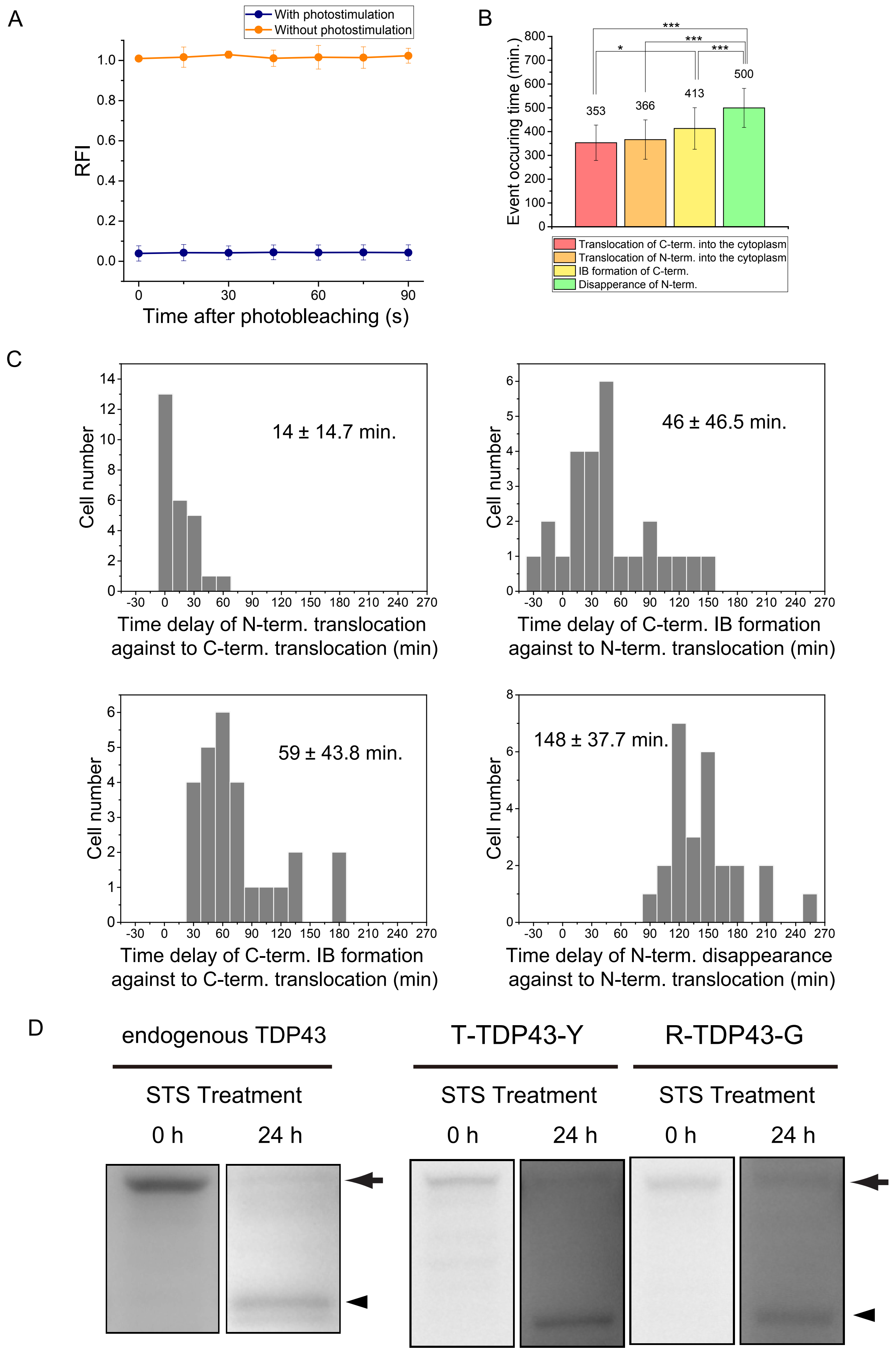

Supplemental Figure 2

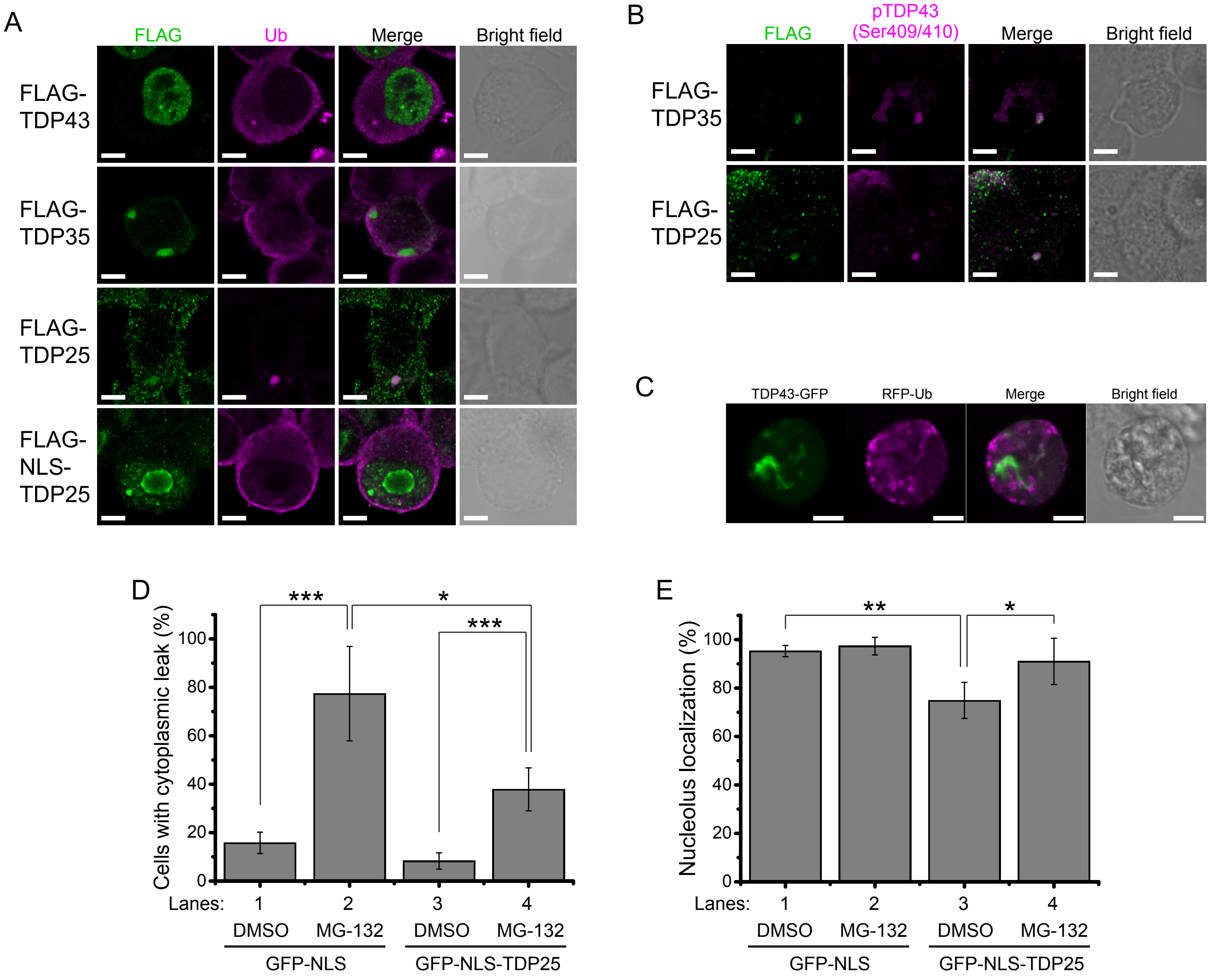

Supplemental Figure 3

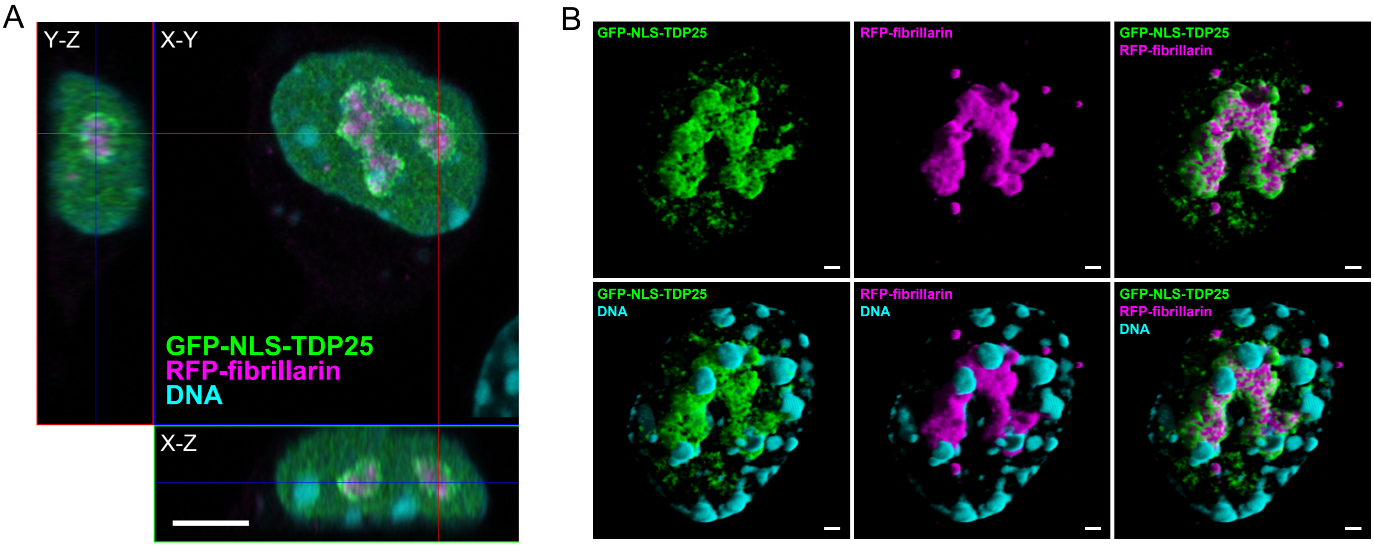

Supplemental Figure 4

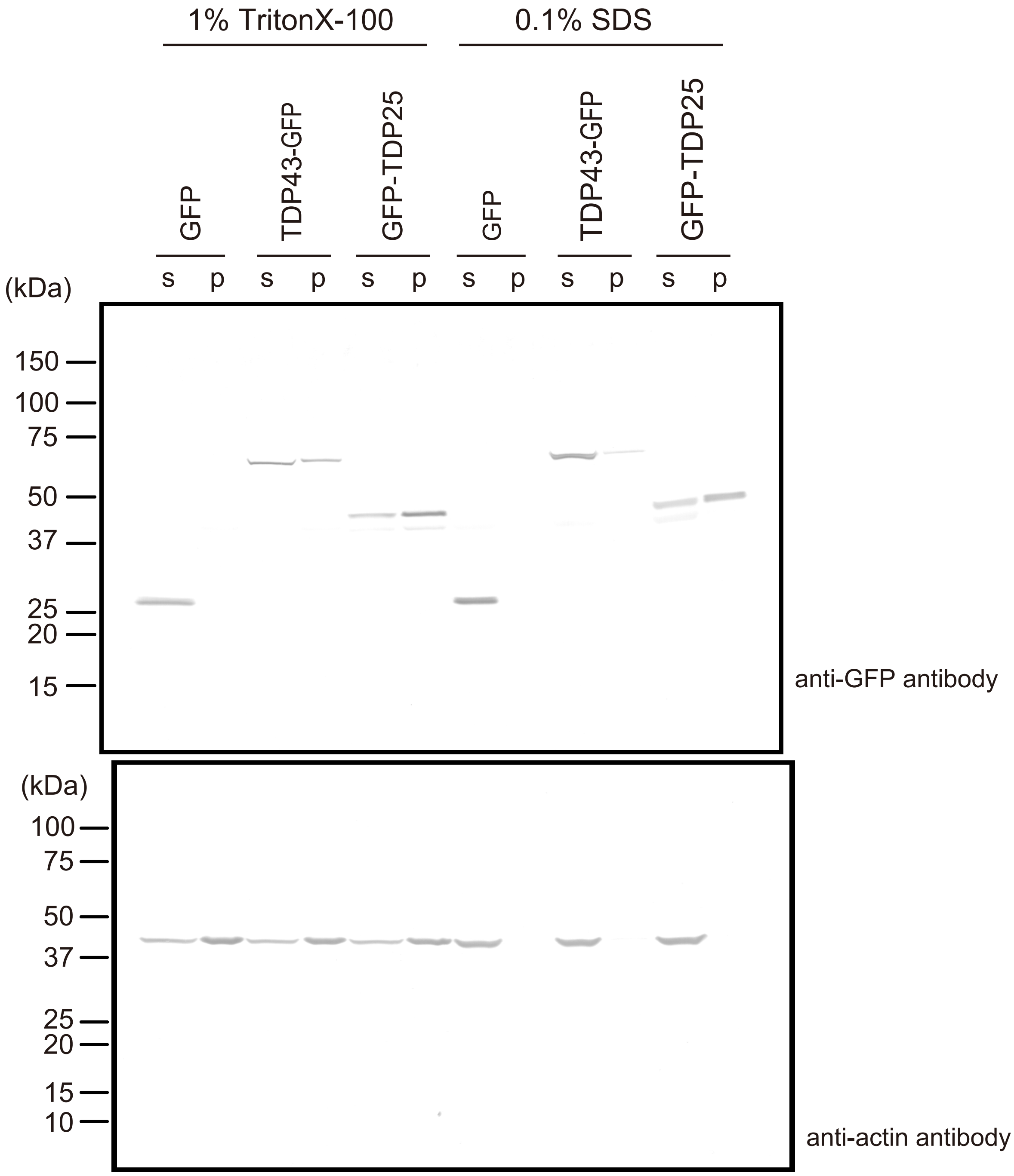

Supplemental Figure 5

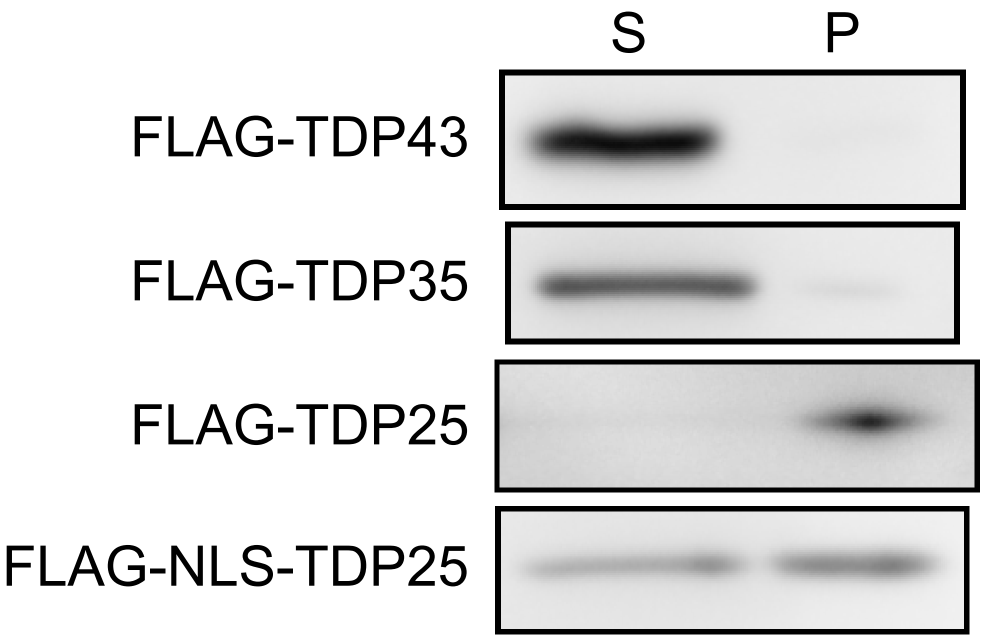

Supplemental Figure 6

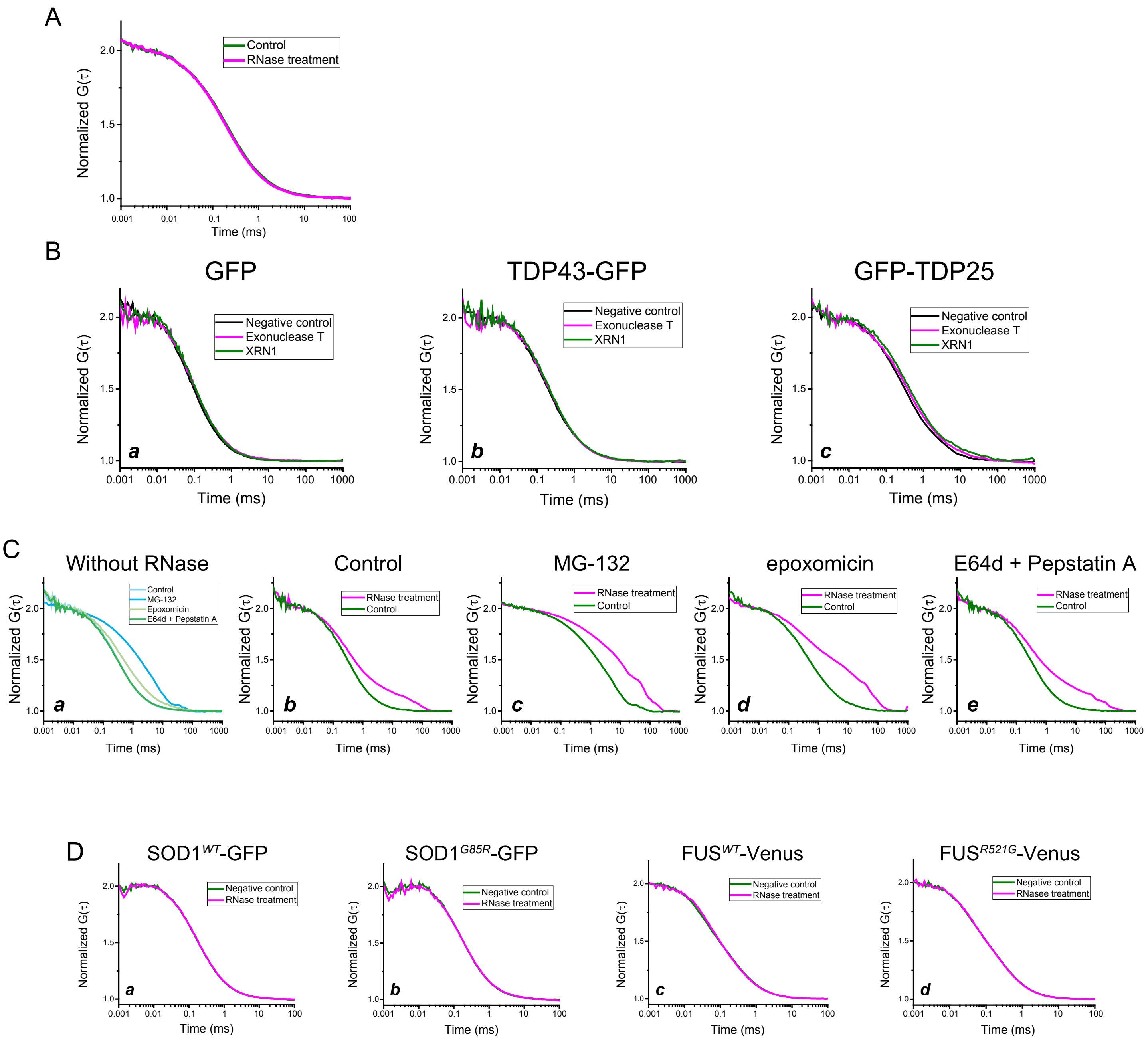

Supplemental Figure 7

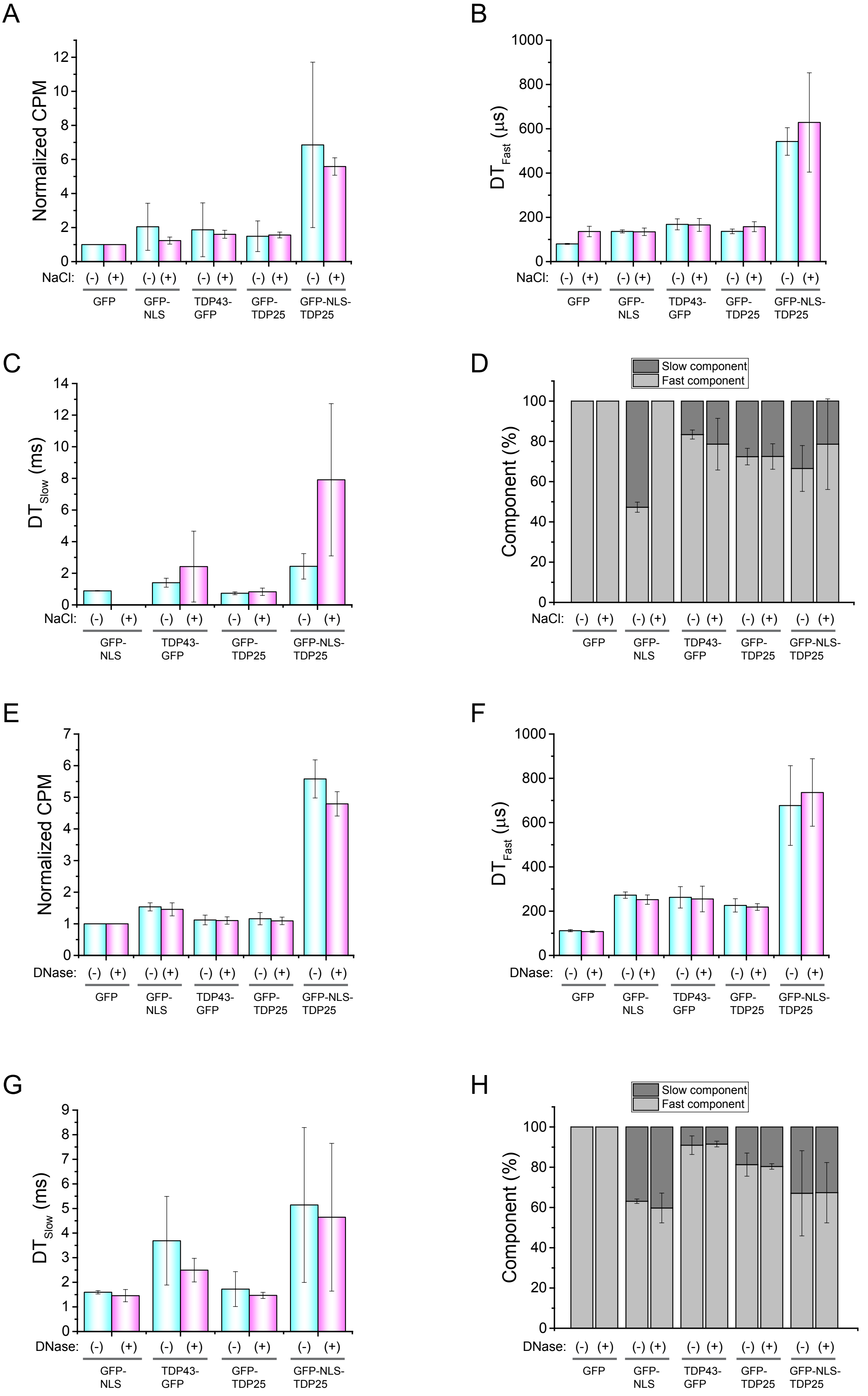

Supplemental Figure 8

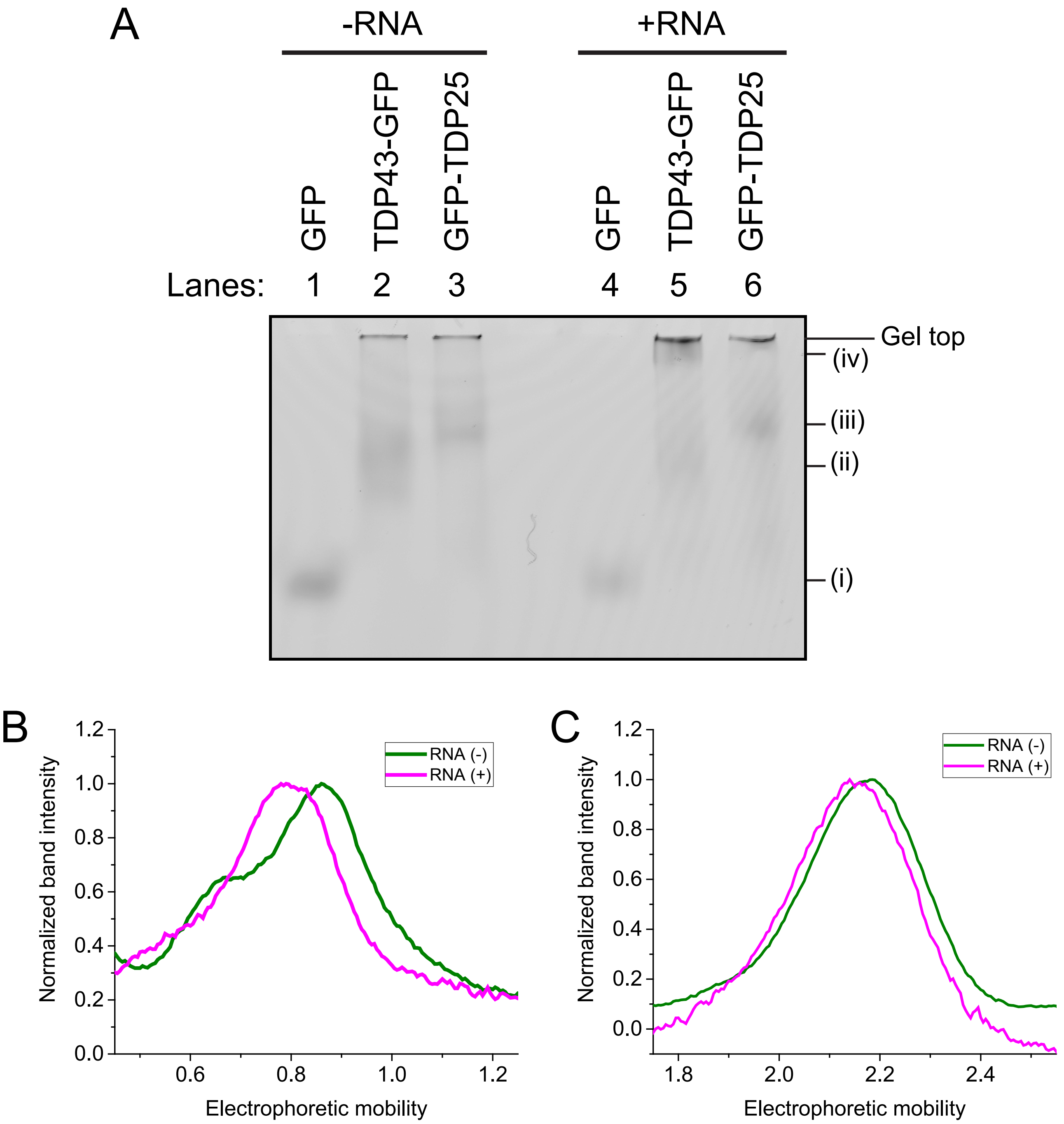

Supplemental Figure 9

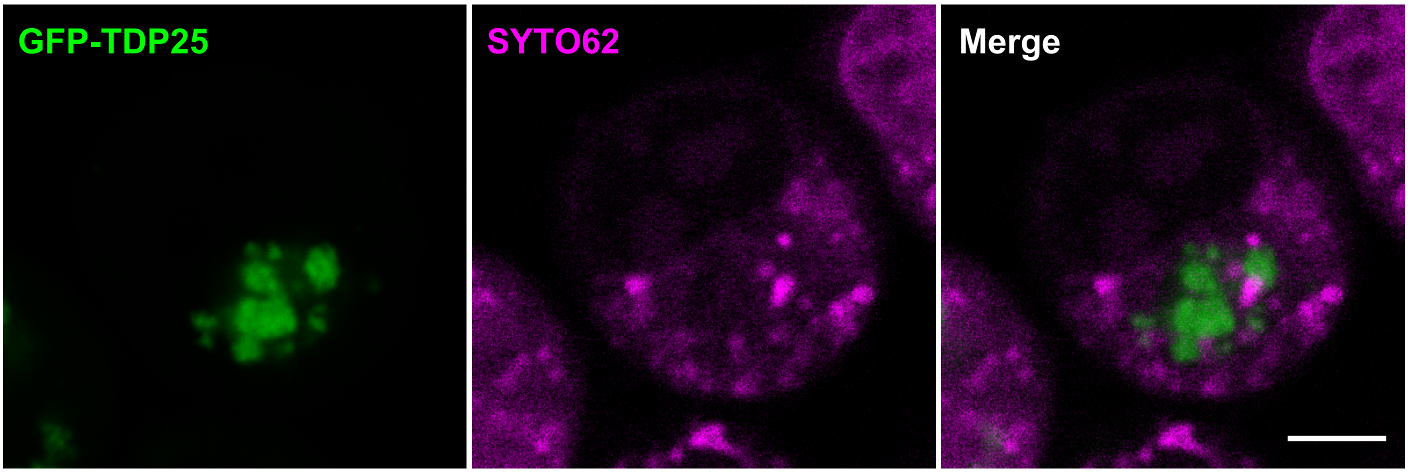

Supplemental Figure 10

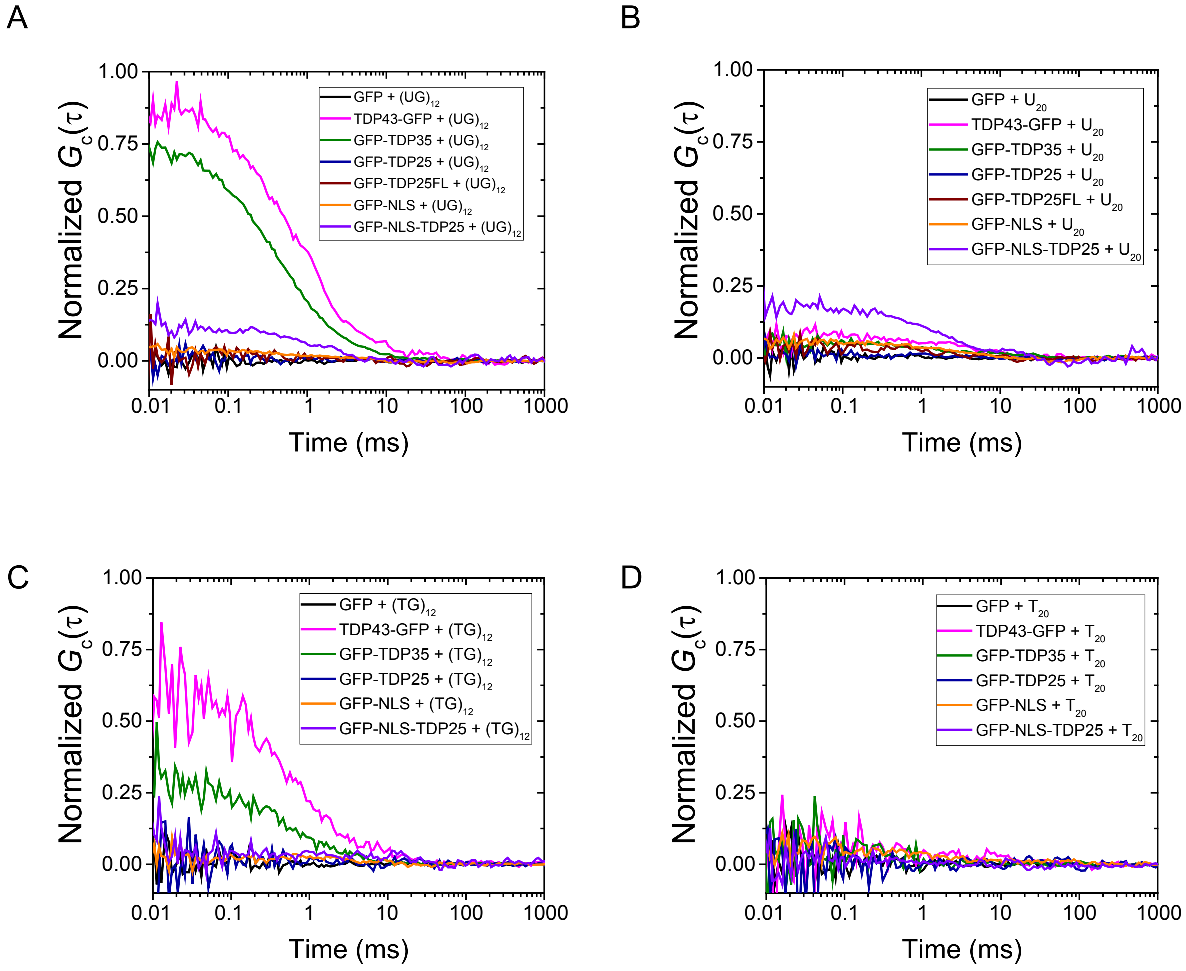

Supplemental Figure 11

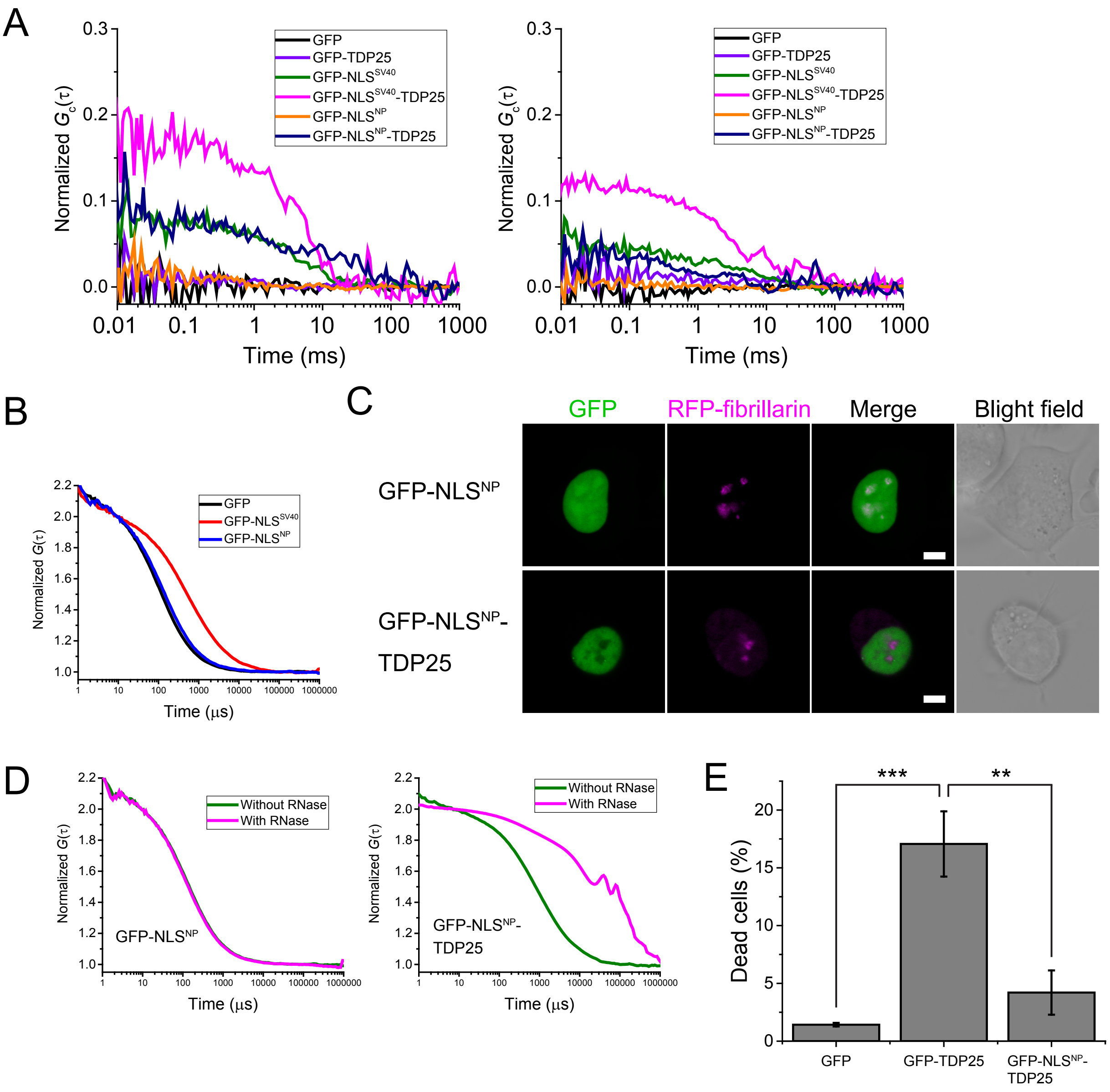

Supplemental Figure 12

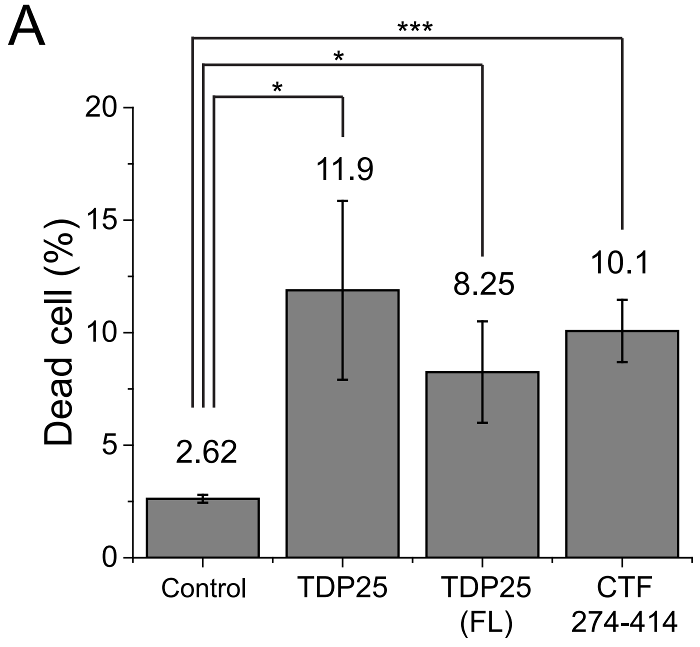

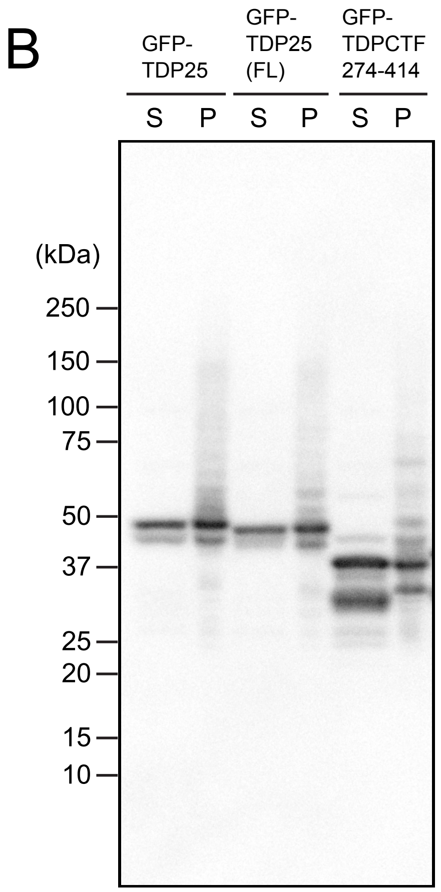

Supplement: Supplementary Information [file srep19230-s1.pdf]
